# Temporal evolution and pathway models of poly(ethylene-terephthalate) degradation under multi-factor accelerated weathering exposures

**DOI:** 10.1371/journal.pone.0212258

**Published:** 2019-02-15

**Authors:** Abdulkerim Gok, Cara L. Fagerholm, Roger H. French, Laura S. Bruckman

**Affiliations:** 1 Department of Materials Science and Engineering, Gebze Technical University, Gebze, Kocaeli, Turkey; 2 SDLE Research Center, Department of Materials Science and Engineering, Case Western Reserve University, Cleveland, Ohio, United States of America; Western Washington University, UNITED STATES

## Abstract

Photolytic and hydrolytic degradation of poly(ethylene-terephthalate) (PET) polymers with different stabilizers were performed under multiple accelerated weathering exposures and changes in the polymers were monitored by various evaluation techniques. Yellowing was caused by photolytic degradation and haze formation was induced by combined effects of photolytic and hydrolytic degradation. The formation of light absorbing chromophores and bleaching of the UV stabilizer additive were recorded through optical spectroscopy. Chain scission and crystallization were found to be common mechanisms under both photolytic and hydrolytic conditions, based on the infrared absorption of the carbonyl (*C* = *O*) band and the *trans* ethylene glycol unit, respectively. The degradation mechanisms determined from these evaluations were then used to construct a set of degradation pathway network models using the network structural equation modeling (netSEM) approach. This method captured the temporal evolution of degradation by assessing statistically significant relationships between applied stressors, mechanistic variables, and performance level responses. Quantitative pathway equations provided the contributions from mechanistic variables to the response changes.

## Introduction

The increase in the global installed photovoltaic (PV) capacity [1] along with module efficiencies [2] and the decrease in the unit cost of electricity generation [3] are promising for the world’s renewable energy needs. While the solar market continues to expand, the reliability of PV systems still remains a challenge [4]. The stability of polymeric components used in PV module encapsulation, such as the encapsulants and backsheets, is of critical importance for service lifetime of PV modules during outdoor deployment. The polymeric backsheets provide electrical insulation for safe operation and act as an environmental barrier to protect cells and electrical components of PV modules. However, environmental stressors present in all real-world climatic zones, such as ultraviolet (UV) light, heat, and humidity, induce physical and chemical degradation in polymers. The failure of backsheets can compromise the electrical insulation and permeation properties and thus lead to performance loss or even dielectric breakdown of PV modules. Backsheet defects [5–8], such as delamination, embrittlement, and discoloration, have been reported as one of the failure mechanisms observed in fielded PV modules [9–11].

Poly(ethylene-terephthalate) (PET) is used in PV backsheets as a core layer because of its high dielectric breakdown strength, but it degrades under real-world conditions [12]. Degradation occurs due to changes associated with the polymer backbone mainly at the ester linkage and results in decreased molecular weight with concomitant changes in morphology leading to deterioration of mechanical properties and thus backsheet failure. Photochemical process involves photolytic chain scissions through Norrish type reactions [13–16]. Under hydrolytic conditions, depending on the degradation environment and degradation byproducts formed, various degradation kinetic schemes have been proposed [17–20]. Stabilizer additives are frequently used to protect the polymers from the long-term effects of environmental stressors and hence extend the service lifetime in real-world conditions; however, these additives can also degrade over time and leave polymers unprotected [21, 22].

In today’s solar market, PV modules’ performance warranty typically guarantee 75% power production at the end of 25 years with 1% loss each year [23–25]. Although standardized qualification tests are used for certification of PV systems or materials, long-term reliability of these systems is still a concern [26–28]. In these lab-based tests, stressors (stress variables) are intensified (i.e., applied in higher than the usual levels seen in real-world conditions) and the observed response is often extrapolated to predict a service lifetime [29]. These tests can be helpful for identifying manufacturing defects and infant mortality in short time frames; however, they do not reflect a realistic environment for actual use conditions and therefore are not capable of estimating long-term performance [30]. The misuse of standardized tests to predict service lifetime of materials was presented by Pickett [31] for the degradation of PET and PC (polycarbonate) polymers under the well-known damp heat (85°C / 85% RH) exposure conditions. Even though PET degraded more rapidly than PC at the test condition, when extrapolated to the ambient end-use temperature (25°C), PET survived longer than PC. Since the activation energies are temperature dependent, the use of the activation energy at 85°C for a lifetime estimation of ambient temperatures caused problems.

The lifetime and degradation science (L&DS) approach has been developed to quantitatively investigate the temporal evolution of degradation mechanisms and pathways using a detailed understanding of domain knowledge and statistical data analytics [32]. Epidemiological studies, in combination with statistically informed study protocols and step-wise observational data, are essential in defining interactions between applied stressors and responses [33, 34]. With this approach, network models of mechanisms and pathways can be realized with appropriate characterizations and failure modes that relate to end-of-life criteria [35, 36]. Due to pass/fail nature of the standardized tests, they lack quantitative information about degradation mechanisms and rates. Our focus is to create degradation pathway network models that can capture the dependence and activation of multiple mechanisms under multiple combined stressors and stress levels using a < Stress|Mechanism|Response > framework [37, 38]. In order to achieve this, network structural equation modeling (netSEM) was developed [39–41] so as to encompass and capture the temporal evolution and multiple mesoscale interactions associated with degradation over lifetime. This method explores the statistically significant relationships between applied stressors and responses and contributing factors to degradation. This can provide a predictive framework [42] for service life prediction [43, 44] by understanding the effective lifetime of a system or material and the fundamental degradation pathways that lead to end-of-life failure.

In this study, a number of lab-based accelerated exposures were applied to realize photolytic and hydrolytic degradation of different PET grades. Several optical and chemical evaluation techniques and exploratory data analysis were conducted to determine the physical and chemical changes in the materials. The netSEM approach was then applied to explore degradation mechanism pathway models using the variables that relate to degradation. Running well-planned experiments and developing statistical models, not subject to bias and irreproducibility, are critical to advance the degradation science of PV modules and materials.

## Experimental methods

### Materials

Three different PET grades were studied in this work: unstabilized (Dupont-Teijin Melinex 454, 75 *μ*m), UV stabilized (Dupont-Teijin Tetoron HB3, 50 *μ*m), and hydrolytically stabilized (Mitsubishi 8LH1, 125 *μ*m). For each grade, samples were cut from a4 size sheets into seven, 3.81 cm by 5.08 cm (1.5 in by 2 in) rectangular, freestanding film replicates. Their chemical compositions and stabilizer contents can be found in S1 Appendix. In the figures, these grades are referred to as Unstab, UVStab, and HydStab, respectively.

### Exposures

In this longitudinal study design [45–48], seven samples from each PET grade were randomly assigned to each of four exposure types. The total exposure time was 1176 hours for each exposure and step-wise evaluations were performed on all samples every 168 hours for seven steps. The samples were taken out of exposures at each exposure step regardless of the exposure cycle the samples were in. The measurements were conducted after the samples had reached room temperature. One sample from each grade was withdrawn from further exposure at each exposure step to allow future evaluations. Table 1 shows the conditions for the four lab-based accelerated exposures used in this work.

**Table 1:** Exposure conditions.

Exposure	Condition
FreezeThaw (IEC 61215-2 MQT 12)	Cyclic heat and humidity (20 hrs at 70°C and 85% RH and 30 min at -40°C)
DampHeat (IEC 61215-2 MQT 13)	Constant heat and humidity (85°C and 85% RH)
HotQUV (ASTM G154 Cycle 4)	Constant heat and UVA light (1.55 W/m^2^ at 340 nm at 70°C)
CyclicQUV (ASTM G154 Cycle 4)	Cyclic heat, humidity, and UV light (8 hrs of UVA light at 1.55 W/m^2^ at 340 nm at 70°C and 4 hrs of condensing humidity at 50°C in dark)

For the heat and humidity exposures (DampHeat and FreezeThaw), based on the IEC 61215 standard [49], Cincinnati Subzero environmental testing chambers (Model ZPH8) were used. For the FreezeThaw exposure, the original temperature of 85°C, stated by the standard, was reduced to 70°C to maintain PET samples below the glass transition temperature during temperature cycling. PV modules are expected to show no more than 8% power degradation, any open circuit and major visual defects, and any changes to the insulation and wet leakage current during 1000 hours of damp heat and 10 cycles of humidity freeze tests.

For the UV light exposures (HotQUV and CyclicQUV), as per the ASTM G154 Cycle 4 [50] standard, Q-Lab QUV accelerated weathering testers (Model QUV/Spray with Solar Eye Irradiance Control) were used. In these testers, UVA-340 fluorescent lamps that emit light between 280 and 400 nm were used at an irradiance level of 1.55 W/m^2^ at 340 nm, approximately 3 times the intensity of air mass (AM) 1.5 solar spectrum [51] at 340 nm. The spectral power distribution of the UVA-340 lamps closely matches solar spectrum at the wavelengths between 280 and 360 nm and thus replicates the UV light damage caused by the natural sunlight. The CyclicQUV exposure is a multi-cyclic and multi-stressor exposure designed to simulate outdoor weathering. It involves alternating sequences of UV light, heat, and condensing humidity, mimicking real-world conditions where materials face morning dew or rain followed by sunlight.

### Performance and mechanistic spectral evaluations

Yellowness index (YI) and haze (%) values were measured using a HunterLab UltrascanPro colorimeter. YI is a measure that describes the change in color of a clear (or white) sample toward yellow as defined by the ASTM E313 standard [52]. Yellowing is mostly caused by the presence of impurities during processing or degradation during use by environmental stressors such as light, heat, and humidity. Haziness is a measure of apparent cloudiness of a sample as defined by the ASTM D1003-13 standard [53]. It is calculated as the percentage of incident light scattered by more than 2.5° in the spectral range from 380 to 780 nm. Haze formation is mostly induced either by bulk scattering due to impurities, inhomogeneities, or crystallinity, or surface scattering due to surface roughness or abrasion.

An Agilent Cary 6000i UV-Vis-NIR spectrometer was used for UV-Vis optical absorbance changes in the degraded samples. The center mount absorbance spectra were taken by using a DRA-1800 diffuse reflectance accessory. Measurements were performed from 250 to 900 nm every 0.50 nm with a scan rate of 112.5 nm/min and a spectral bandwidth of 4.00 nm. All the spectra were initially corrected for zero absorbance between 600 and 800 nm and then normalized for the sample thickness to get Abs/cm metric.

Chemical changes in the degraded samples were evaluated using an Agilent 630 FTIR spectrometer with an attenuated total reflectance (ATR) accessory utilizing a single reflection with nominal angle of 45°. The spectra were taken between 650 and 4000 cm^−1^ every 2 cm^−1^ resolution with eight background and sample scans. Baseline correction was first applied using a polynomial fitting [54, 55]. Normalization with the peak intensity of the IR band at 1410 cm^−1^ was then applied in order to eliminate inconsistency in spectra caused by the contact between the sample and ATR crystal. The aromatic skeletal stretching band at 1410 cm^−1^ is known as the internal IR reference in PET as it is insensitive to polarization, conformation, and orientation [56–59].

Spectral data points extracted from both UV-Vis and IR spectroscopic techniques were used as mechanistic variables in the netSEM analysis.

Since these optical and chemical spectroscopic techniques mostly provide indirect measures of physical and chemical properties, direct measures of degradation characteristics were conducted via several evaluation techniques in order to confirm the validity of the spectral observations. Technical details and results can be found in S2 Appendix for crystallinity measurements via Differential Scanning Calorimetry (DSC), S3 Appendix for intrinsic viscosity and molecular weight measurements, and S4 Appendix carboxyl end group (CEG) content analysis.

### Data analytics

A L&DS approach is applied to find degradation mechanisms and contributing factors to degradation. The relationships between stressors (*S*), system level mechanistic responses (*M*), and performance level responses (*R*) are determined via network structural equation modeling (netSEM) approach [39–41].

In this < *S*|*M*|*R* > study, the experimental results from optical and chemical spectral evaluations are the basis of system level mechanistic (*M*) variables with exposure time as a proxy to stressors (*S*), and depending on the exposure condition, i.e., photolysis or hydrolysis, yellowness index (YI) and haze (%) are selected as the performance level responses (*R*). When compared to traditional SEM, netSEM is generalized to fit non-linear functional forms (i.e., simple quadratic, quadratic, logarithmic, and exponential) and linear change point models that are seen in physical and chemical degradation processes. It is also supervised with domain knowledge that is observed in existing models in the literature. In the SEM approach, the coupling strength between two variables is represented by *β*_*ij*_, aka the coefficient vector of the fitting model, that predicts variable *i* from *j*. The functional form between two variables is chosen from a pool of candidates and the best model is achieved based on the adjusted R^2^ values. Using step-wise regression modeling, all of the univariate relationships are rank-ordered sequentially and mapped into a pathway model that involves the contributing factors to degradation [60].

Pathway diagrams are then obtained using the pair-wise relationships between stressors, mechanisms, and responses. In these diagrams, arrows represent relationships between variables with statistical metrics provided along the connection lines. In order to rank these connection lines, two adjusted R^2^ cutoff values of 0.5 and 0.75 are selected. Strong relationships (adjusted R^2^ ≥ 0.75) are shown with thick solid lines, moderate relationships (0.75 > adjusted R^2^ ≥ 0.5) with thin solid lines, and weak relationships (adjusted R^2^ < 0.5) with dotted lines.

Because this approach only determines univariate relationships, multi-step (mechanistic) pathways are derived by substituting one mechanistic variable equation into another. For example, to determine the multi-step path of “*S* → *M* → *R*”, first, equations for the “*S* → *M*” and “*M* → *R*” relationships are obtained; and then, “*M*” in the first model is substituted into the second model to achieve the “*M*” dependent multi-step path of “*S* → *R*”. Plotting these mechanistic equations over the applied exposure time shows the unique response with time and how the system level mechanisms contribute to the overall behavior. Quantitative comparison between the direct < *S*|*R* > and the multi-step < *S*|*M*|*R* > pathways is performed using the root mean square error (RMSE), which is the difference between the observed response and predicted response, as shown in Eq 1. In this equation, the raw observation is (*y*) and the predicted response ($\hat{y}$) is calculated by using the fitted model. The smaller an RMSE value, the closer observed response and predicted values are.

$$\begin{array}{c} RMSE=\sqrt{\frac{1}{n}\sum_{i=1}^{n}{\left(y_{i}-\hat{y_{i}}\right)}^{2}} \end{array}$$ (1)

## Results

### Performance and mechanistic evaluations

#### Yellowness index (YI) and haze (%)

**Fig 1:**
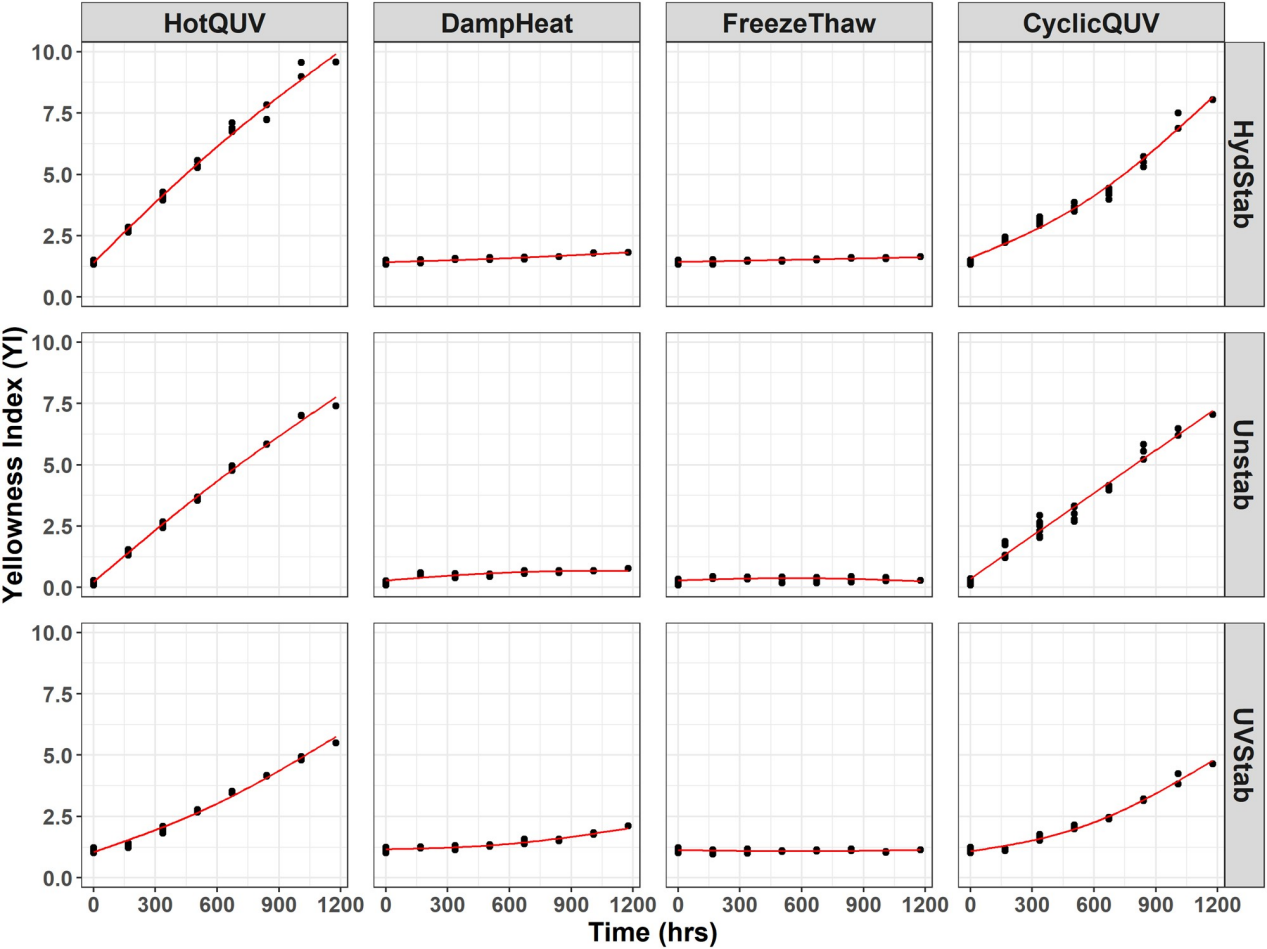
Cross panel figure for the change in YI with exposure time for each type of PET grade. Note that the exposures are shown as columns and PET grades are shown as rows in the figure. Each exposure step (168 hours) corresponds to one week of exposure and the total exposure time (1176 hours) of seven steps corresponds to seven weeks of exposure. Regression lines are obtained through pathway equations from the netSEM analysis and represent the best fit for each relationship based on adjusted R^2^ values.

Fig 1 shows a cross panel figure for the change in YI in all grades and exposure types. There is a marked increase in YI with the HotQUV and CyclicQUV exposures and negligible change with the DampHeat and FreezeThaw exposures. This shows that UV light induces more yellowing and thus more degradation when compared to non-UV light exposures. The effect of the UV stabilizer under the HotQUV and CyclicQUV exposures is apparent from the change points observed after the first exposure step. During this “induction” period, YI exhibits only slight change, so the mechanism is suppressed, retarded by the stabilizer, but after the stabilizer has been consumed, a sharp increase is realized.

**Fig 2:**
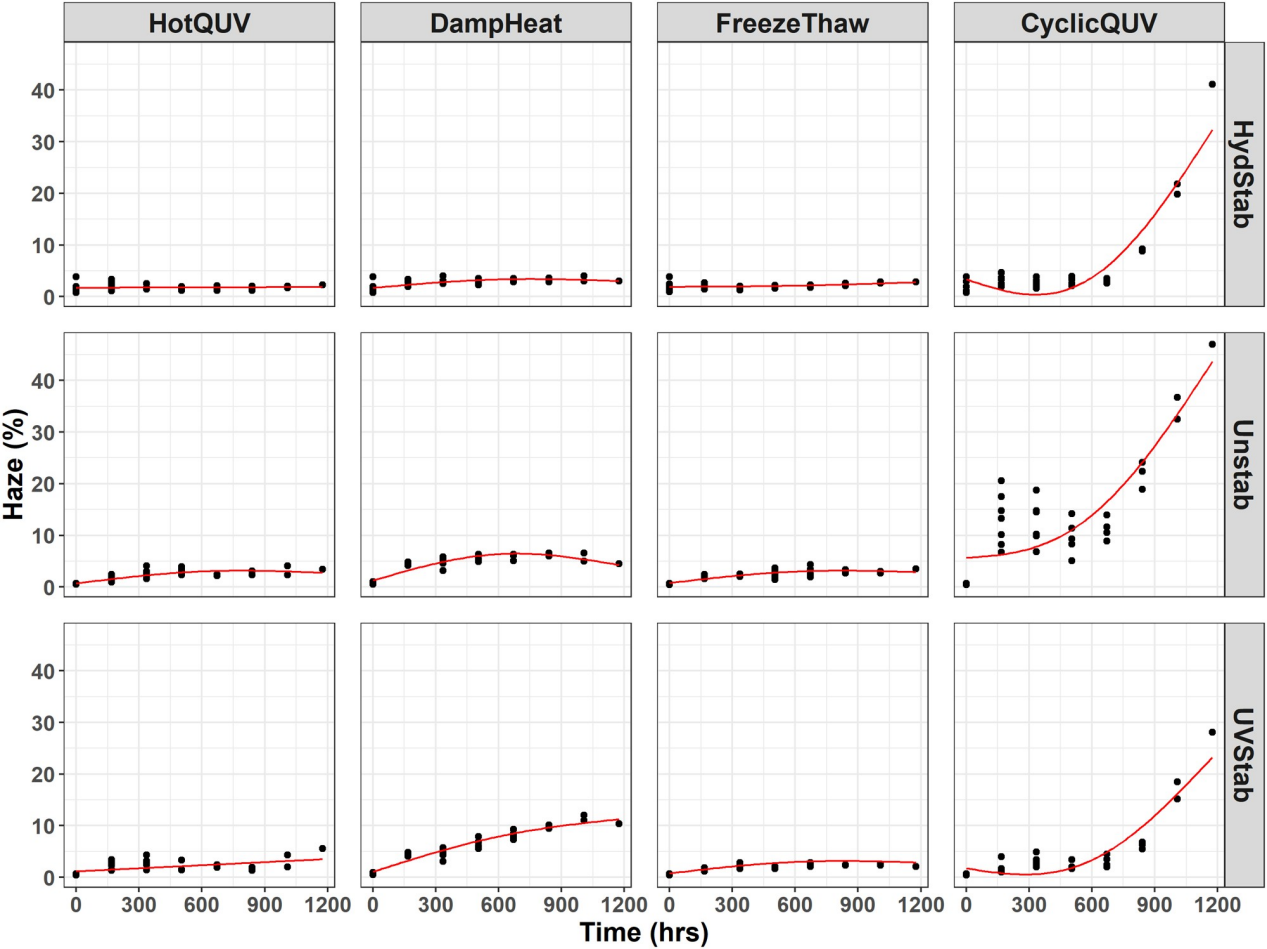
Cross panel figure for the change in haze (%) with exposure time. Note that the exposures are shown as columns and PET grades are shown as rows in the figure. Each exposure step (168 hours) corresponds to one week of exposure and the total exposure time (1176 hours) of seven steps corresponds to seven weeks of exposure. Regression lines are obtained through pathway equations from the netSEM analysis and represent the best fit for each relationship based on adjusted R^2^ values.

Fig 2 shows the temporal evolution of haze development in all grades and exposures with time. Moisture induced hazing is noticeable in all PET grades with the high humidity exposures, especially in CyclicQUV. The protective effect of the hydrolytic stabilizer is questionable. It provides stabilization to some degree in comparison to the unstabilized grade, but is not very effective under these exposure conditions and times. In spite of the high level yellowing, the HotQUV exposure with strong UV light content did not cause any hazing. Haze formation is mainly activated by hydrolytic degradation rather than photolytic, but cyclic temperature can play a significant role. Between-sample variance is seen to be large compared to the YI development due to large measurement uncertainty. This infers that haze formation is more likely a random process, i.e., localized, in lieu of uniform across sample volume as observed with yellowing.

Since no significant changes in YI and haze (%) values were obtained under the DampHeat and FreezeThaw exposures, the discussion of these two will be excluded from further sections.

#### UV-Vis absorbance

**Fig 3:**
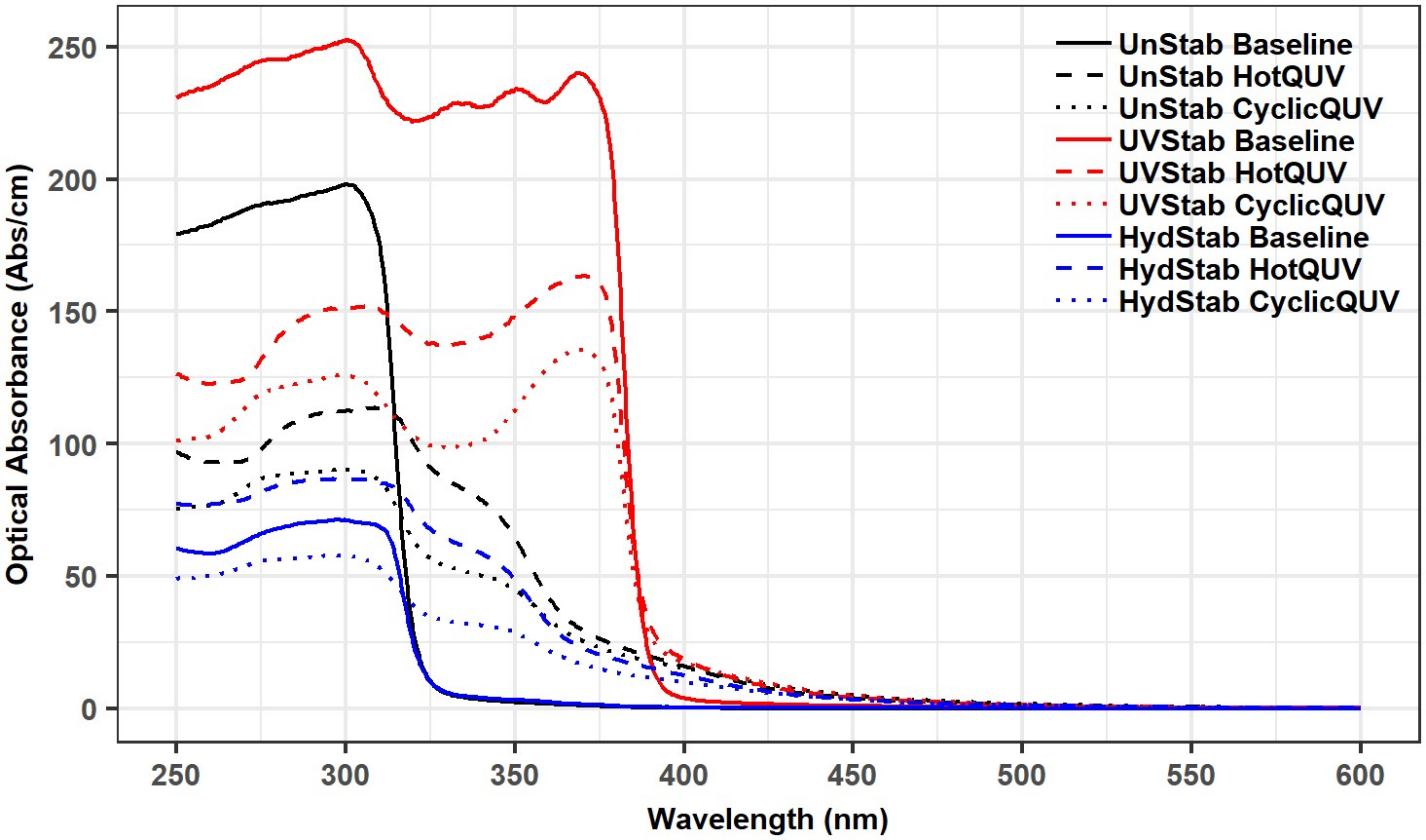
UV-Vis Abs/cm spectra before and after degradation.

The formation of coloring and fluorescing chromophores and the depletion of the UV stabilizer were examined via UV-Vis absorbance spectroscopy. Spectral changes can be seen in Fig 3 only for the unexposed baseline and very last exposed samples of the three PET grades under the HotQUV and CyclicQUV exposures.

**Fig 4:**
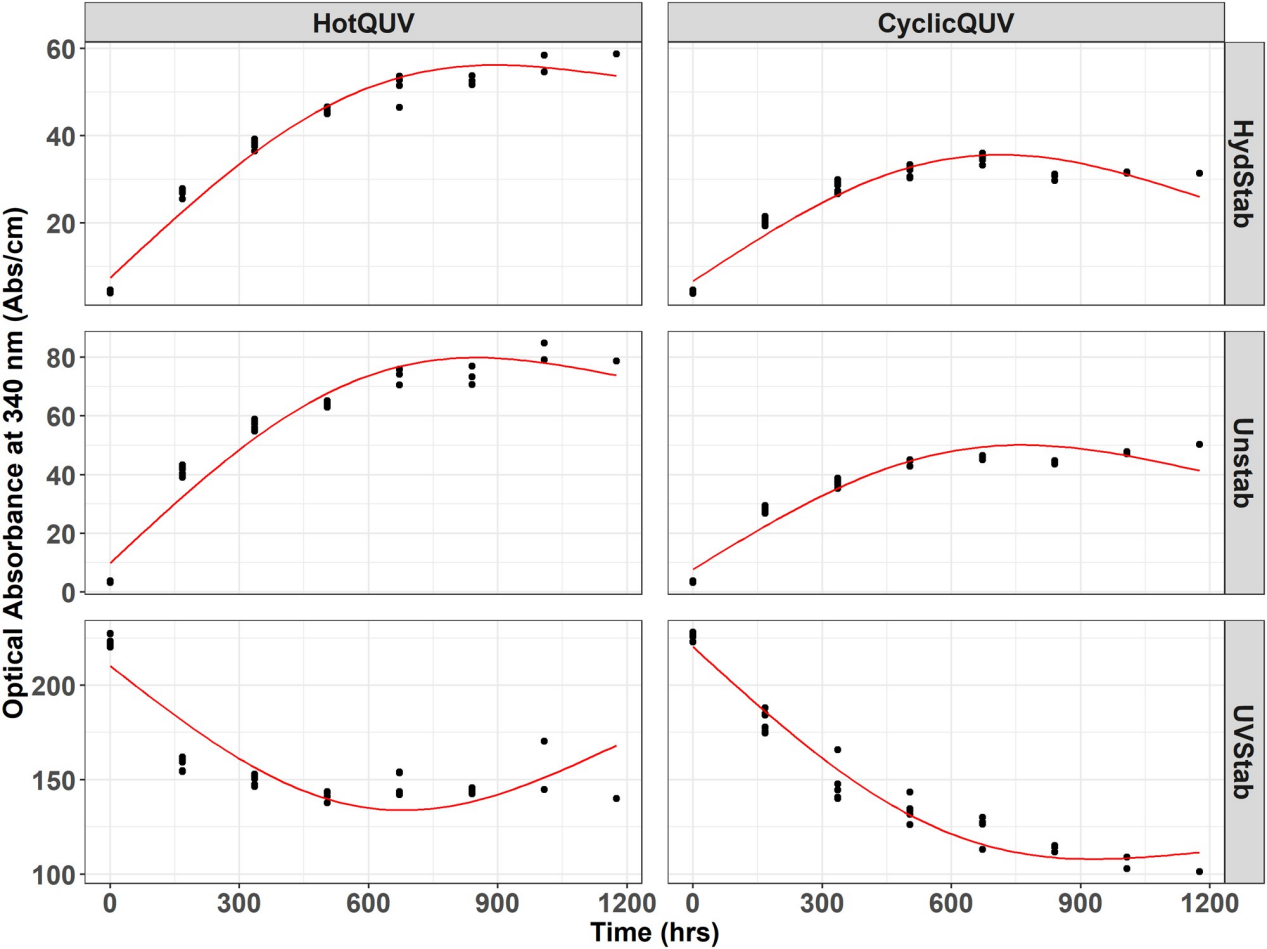
Cross panel figure for the change in UV-Vis Abs/cm at 340 nm with exposure time. Note that the exposures are shown as columns and PET grades are shown as rows in the figure. Each exposure step (168 hours) corresponds to one week of exposure and the total exposure time (1176 hours) of seven steps corresponds to seven weeks of exposure. Regression lines are obtained through pathway equations from the netSEM analysis and represent the best fit for each relationship based on adjusted R^2^ values.

Point in time data were then extracted from spectra at 340 nm and plotted as a function of time for each grade and exposure type as shown in Fig 4. The optical absorbance at 340 nm refers to the formation of hydroxyterephthalate units [61–63] as light absorbing chromophores. For the unstabilized and hydrolytically stabilized grades, the increased absorbance at 340 nm is evident, indicating the formation of light absorbing chromophores. The magnitude of increase is higher in the HotQUV exposure than that in the CyclicQUV exposure due to the greater fraction of photo-dose content of the HotQUV exposure. However, in the UV stabilized grade, the decreased absorbance at 340 nm refers to the depletion of the UV stabilizer whose presence is evident in Fig 3 by an absorption cutoff at 400 nm, and absorption peaks at 330, 340, and 365 nm. When comparing the two exposures, the effect of humidity in the CyclicQUV exposure on the depletion of the UV stabilizer is seen with a larger decrease despite the lower level of photo-dose in this exposure. It is to be noted that all grades under both exposures exhibit a change point model with two (or even three) regimes which were successfully captured by the netSEM analysis.

#### IR absorbance

**Fig 5:**
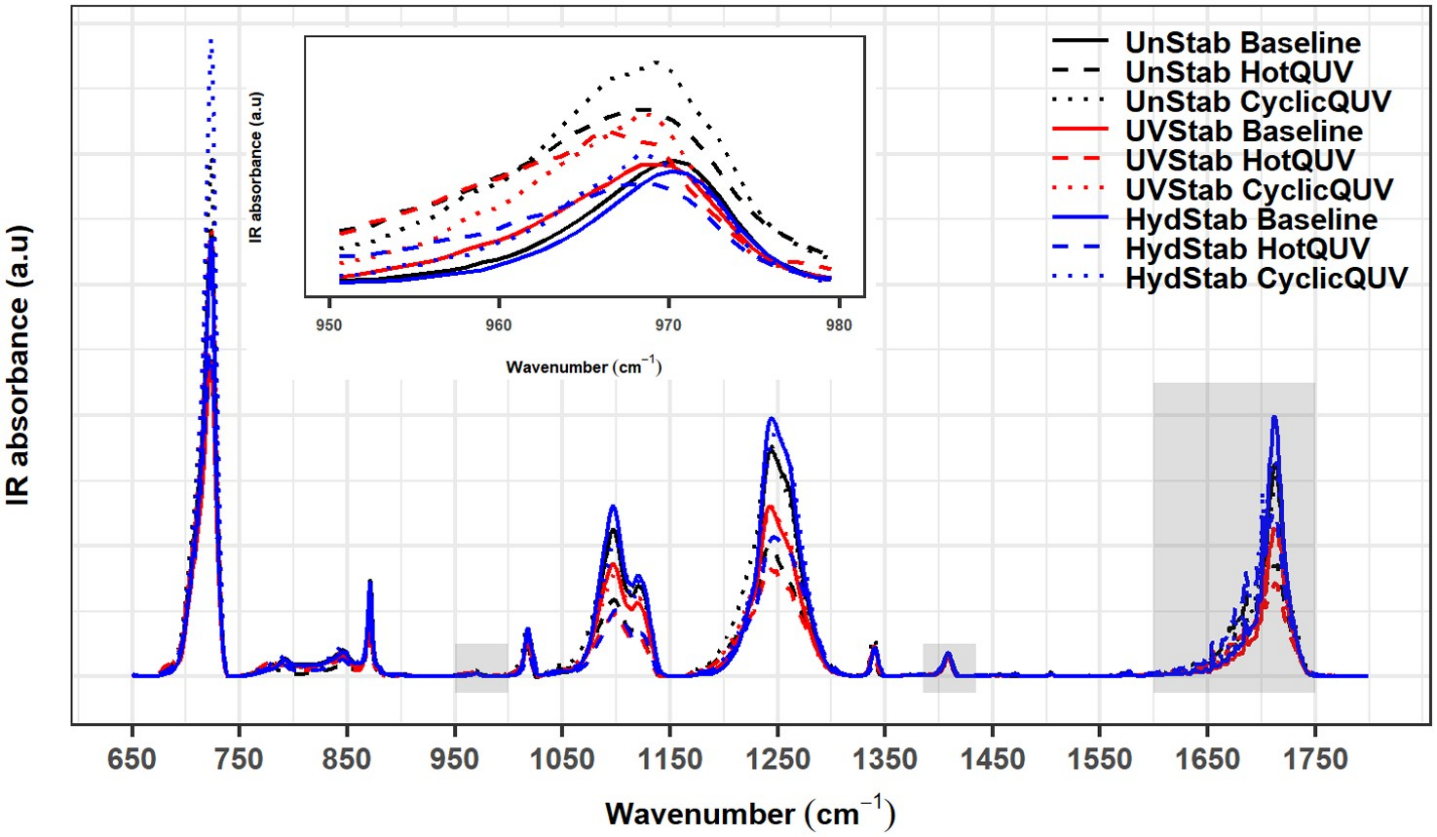
FTIR-ATR spectra before and after degradation. Carbonyl and *trans* ethylene glycol regions and the normalization band are highlighted in rectangles. The inset spectra is the zoomed *trans* ethylene glycol region at 975 cm^−1^.

**Fig 6:**
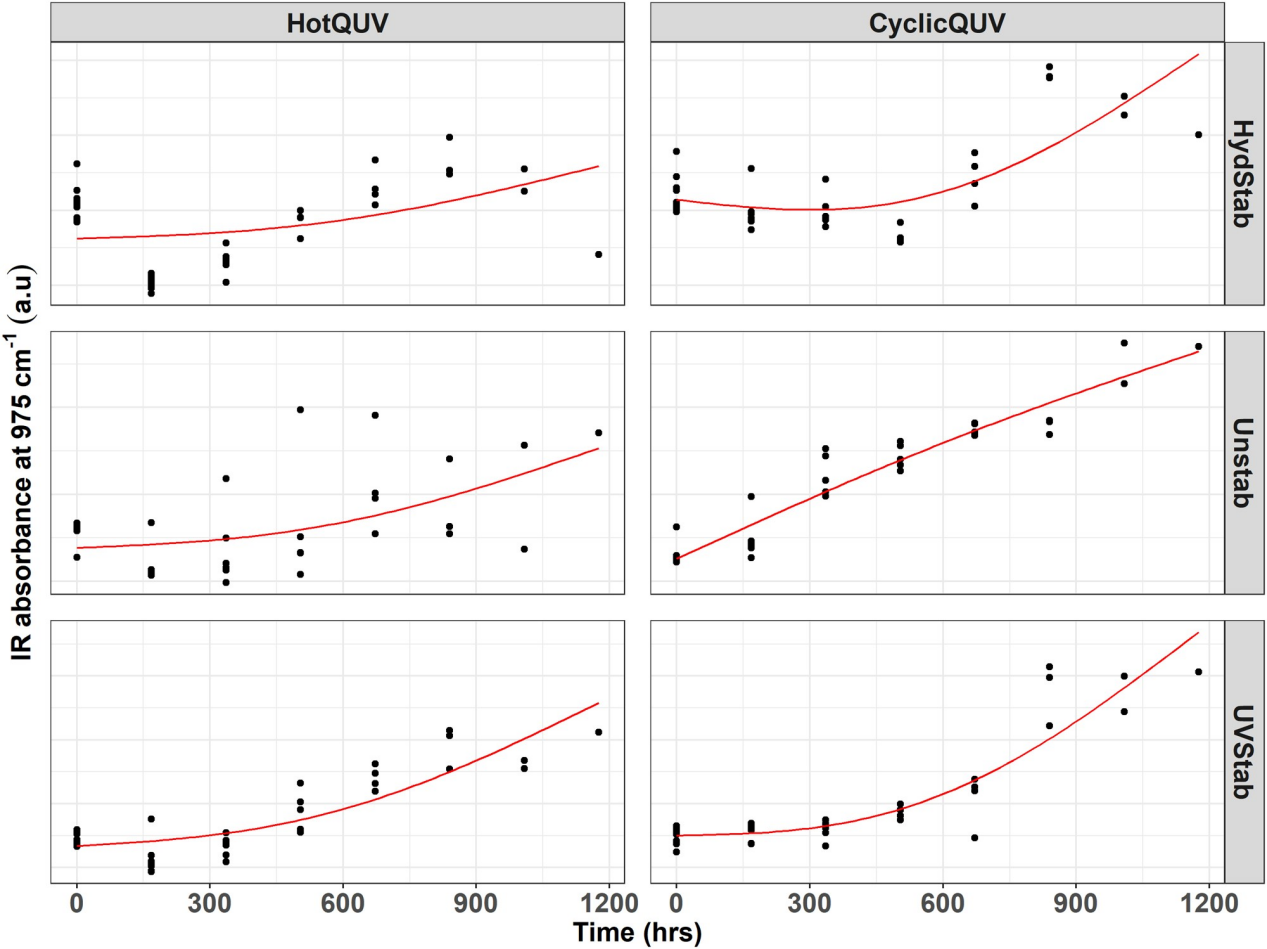
Cross panel figure for the change in IR absorption at 1675 cm^−1^ with exposure time. Note that the exposures are shown as columns and PET grades are shown as rows in the figure. Each exposure step (168 hours) corresponds to one week of exposure and the total exposure time (1176 hours) of seven steps corresponds to seven weeks of exposure. Regression lines are obtained through pathway equations from the netSEM analysis and represent the best fit for each relationship based on adjusted R^2^ values.

**Fig 7:**
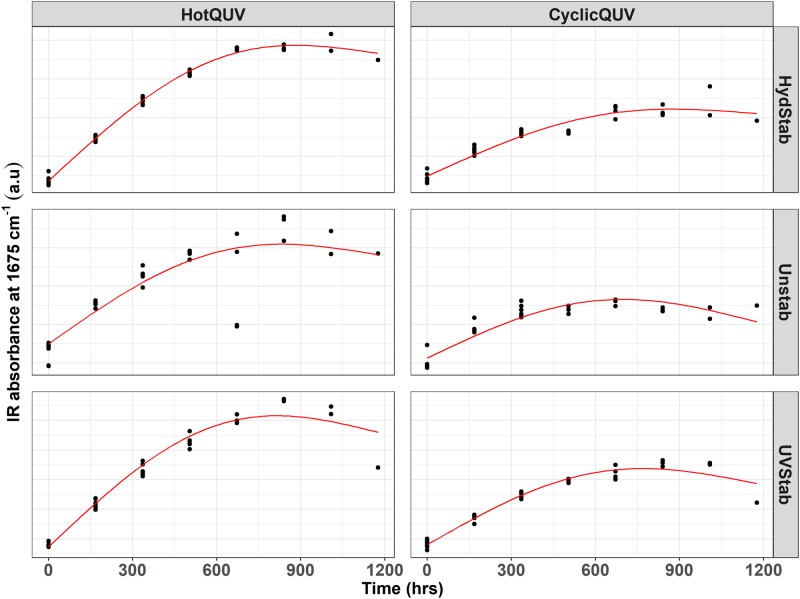
Cross panel figure for the change in IR absorption at 975 cm^−1^ with exposure time. Note that the exposures are shown as columns and PET grades are shown as rows in the figure. Each exposure step (168 hours) corresponds to one week of exposure and the total exposure time (1176 hours) of seven steps corresponds to seven weeks of exposure. Regression lines are obtained through pathway equations from the netSEM analysis and represent the best fit for each relationship based on adjusted R^2^ values.

During photolytic and hydrolytic degradation, similar responses are reported as both degradation mechanisms proceed via cleavage of the ester bond in the polymer chain leading to chain scission, molecular weight loss, and changes in the morphology. Chemical and structural changes were investigated via FTIR-ATR spectroscopy and an example spectral plot is provided in Fig 5 only for the unexposed baseline and very last exposed samples of the three PET grades under the HotQUV and CyclicQUV exposures. Spectral points that relate to changes in the carbonyl band region at 1675 cm^−1^ for chain scission behavior and the *trans* oxy-ethylene (*O* − *CH*_2_) band of the ethylene glycol unit at 975 cm^−1^ for change in morphology, i.e., aging induced crystallization, were then extracted. These data points are plotted as a function of time for each grade and exposure type as shown in Figs 6 and 7, respectively.

For chain scission (i.e., formation of carboxyl end groups), the increased IR absorption intensity at 1675 cm^−1^ is clearly seen in all grades and exposure types. The HotQUV exposure causes more increase over time probably due to the higher photo-dose and heat content, but no distinct differences are observed between the PET grades. For all cases, the slowed down degradation is evident at the extent of exposure. The two mechanisms by which the formation of CEGs occurs during these exposures can be summarized as follows [64]: 1) Hydrolysis results in the scission of polymer chains at the ester linkage, leading to the formation of carboxylic acid and hydroxyl end groups. These acids can also cause an autocatalytic effect in hydrolytic reactions by acting as catalysts and thus promote further degradation. 2) Photodegradation (Norrish type II) occurs by an intramolecular hydrogen abstraction mechanism, leading to rearrangement into a six-membered intermediate ring structure, and with further degradation, carboxyl and vinyl end groups are created.

The aging induced crystallinity upon degradation is important as it relates to mechanical performance such as brittleness and tensile strength of polymers. Because crystalline structure is impermeable to water and oxygen diffusion, initial crystallinity has an enormous effect on stability of the polymers. For the change in morphology, the evolution of the *trans* band suggests that the aging induced crystallization occurred in all grades under both exposure conditions with smaller variance between repeated measurements under the CyclicQUV exposure, as seen in Fig 7. The increased intensity suggests that the CyclicQUV exposure with the addition of humidity led to more crystallization than the HotQUV exposure. Direct crystallinity measured via DSC is included in S2 Appendix to support these observations.

### Degradation pathway models: netSEM analysis of PET degradation

In this section, netSEM pathway diagrams under the HotQUV and CyclicQUV exposures are presented for yellowing of the UV stabilized grade due to its complex degradation with the addition of UV stabilizer. In these diagrams, *Time* is the main stressor as a proxy to exposure conditions and *YI* is the performance level response variable. Mechanistic variables are *ChainScission* for chain scission measured through the IR absorption of the carbonyl band region at 1675 cm^−1^, *Crystallinity* for crystallinity measured through the IR absorption of the *trans* oxy-ethylene (*O* − *CH*_2_) band of the ethylene glycol unit at 975 cm^−1^, *FundAbsEdge* is the fundamental absorption edge measured through the UV-Vis Abs/cm at 312 nm, and *UVStabBleach* is the UV stabilized bleaching measured through the UV-Vis Abs/cm at 340 nm. Pathway curves associated with direct and multi-step (mechanistic) pathways are also provided with RMSE values for each pathway to see the predictive powers of the models. The diagrams for the two other grades on both yellowing and haze formation and their mechanistic pathway curves can be found in S2 through S9 Figs.

**Fig 8:**
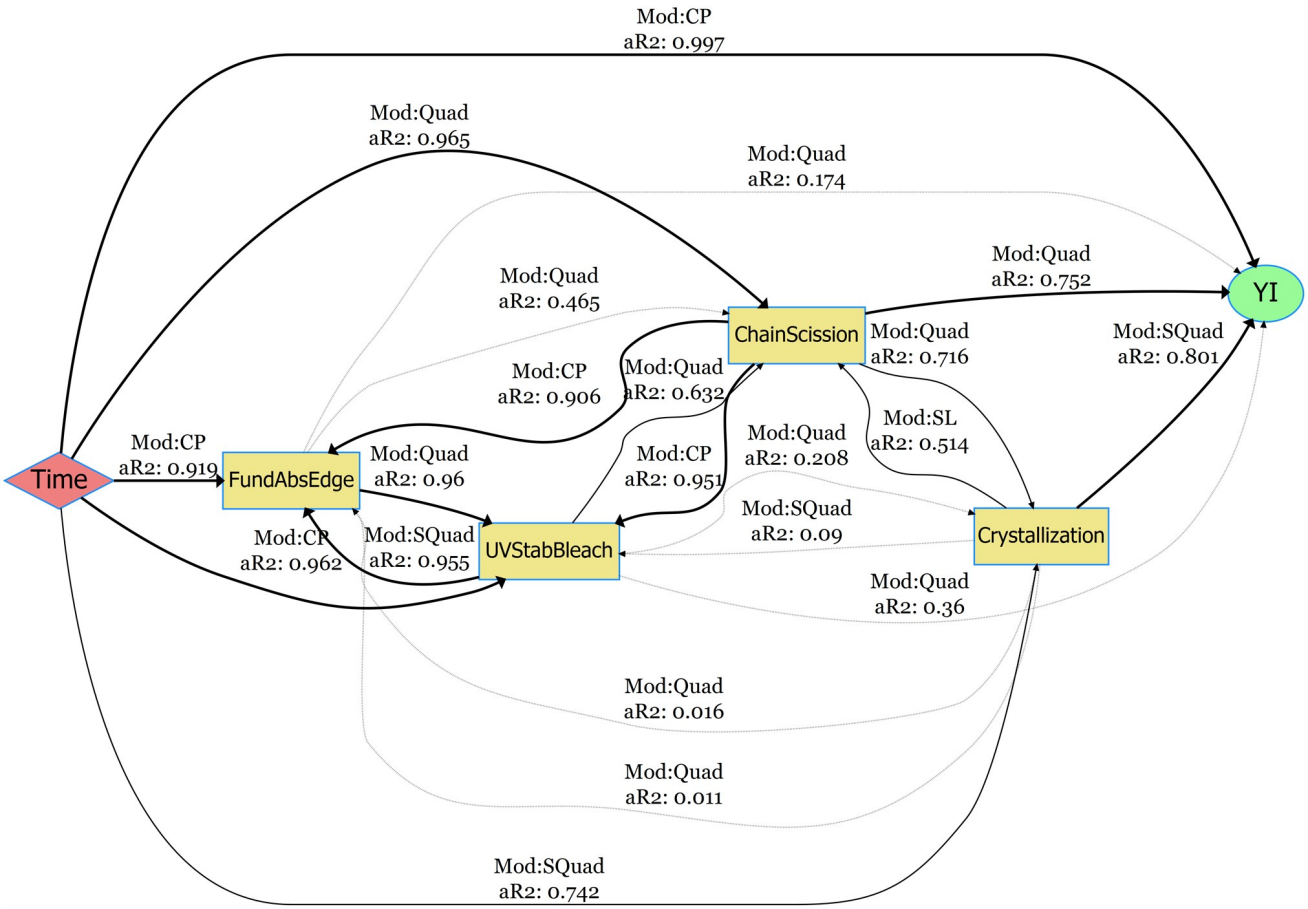
netSEM pathway diagram for the yellowing of UV stabilized grade under the HotQUV exposure. The fitting models (Mod) and the adjusted R^2^ values (aR2) for each relationship are given along the connection lines. The models are SL (simple linear), SQuad (simple quadratic), Quad (quadratic), Exp (exponential), Log (logarithmic), CP (change point), and nls (non-linear least squares regression).

The pathway diagram for the UV stabilized grade under the HotQUV exposure can be seen in Fig 8. The effect of photodegradation is seen with the strong relationship between YI and time with a change point model with a perfect fit. Only two mechanistic variables are acting strongly on YI with adjusted R^2^ values greater than 0.75: crystallization with simple quadratic model and chain scission with quadratic model. The role of UV stabilizer bleaching is not significant on YI even though its consumption with time is shown with a strong relationship. However, the relationship between chain scission and UV stabilizer bleaching suggest an alternative path for YI formation with the UV stabilizer’s consumption.

**Fig 9:**
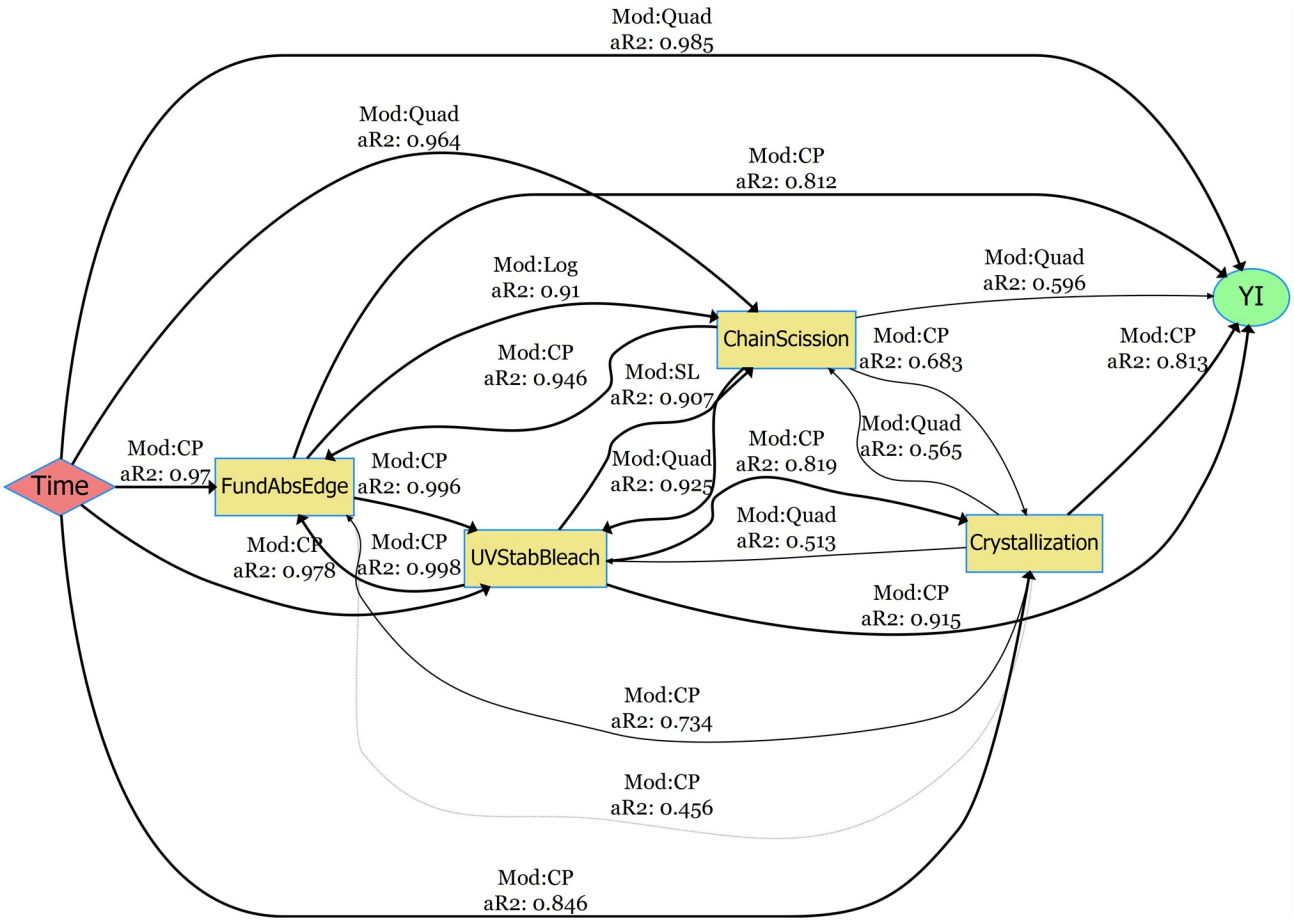
netSEM pathway diagram for the yellowing of UV stabilized grade under the CyclicQUV exposure. The fitting models (Mod) and the adjusted R^2^ values (aR2) for each relationship are given along the connection lines. The models are SL (simple linear), SQuad (simple quadratic), Quad (quadratic), Exp (exponential), Log (logarithmic), CP (change point), and nls (non-linear least squares regression).

Fig 9 is the pathway diagram for the CyclicQUV exposure. All of the mechanistic variables are now strongly linked to YI. This suggests that the moisture coupled with UV light activated all of the mechanistic degradation routes that were not affected strongly under the HotQUV exposure. Interestingly, the bleaching of the UV stabilizer now acts strongly on YI in the CyclicQUV exposure, although it was weakly related in the HotQUV exposure. This could be because of moisture enhanced reactions that might have an accelerated effect on the degradation and cause both polymer and UV stabilizer to degrade. The chemical signals of chain scission and crystallization are also correlated with both time and YI, but at a reduced rate for chain scission compared to that in the HotQUV exposure.

The pathway diagram for the haze formation under the CyclicQUV exposure is provided in S1 Fig. The relationship between time and haze (%) is depicted with a change point model and a high power of fitting. Among the mechanistic variables, only the UV stabilizer bleaching is found to be strongly correlated with both time and haze formation. Crystallization and fundamental absorption edge exhibit similar trends: strongly linked with time, but moderately related to hazing. Since haze formation is directly associated with changes in crystallization, this relationship is predictable, but would be expected to have a greater impact on haze formation.

**Fig 10:**
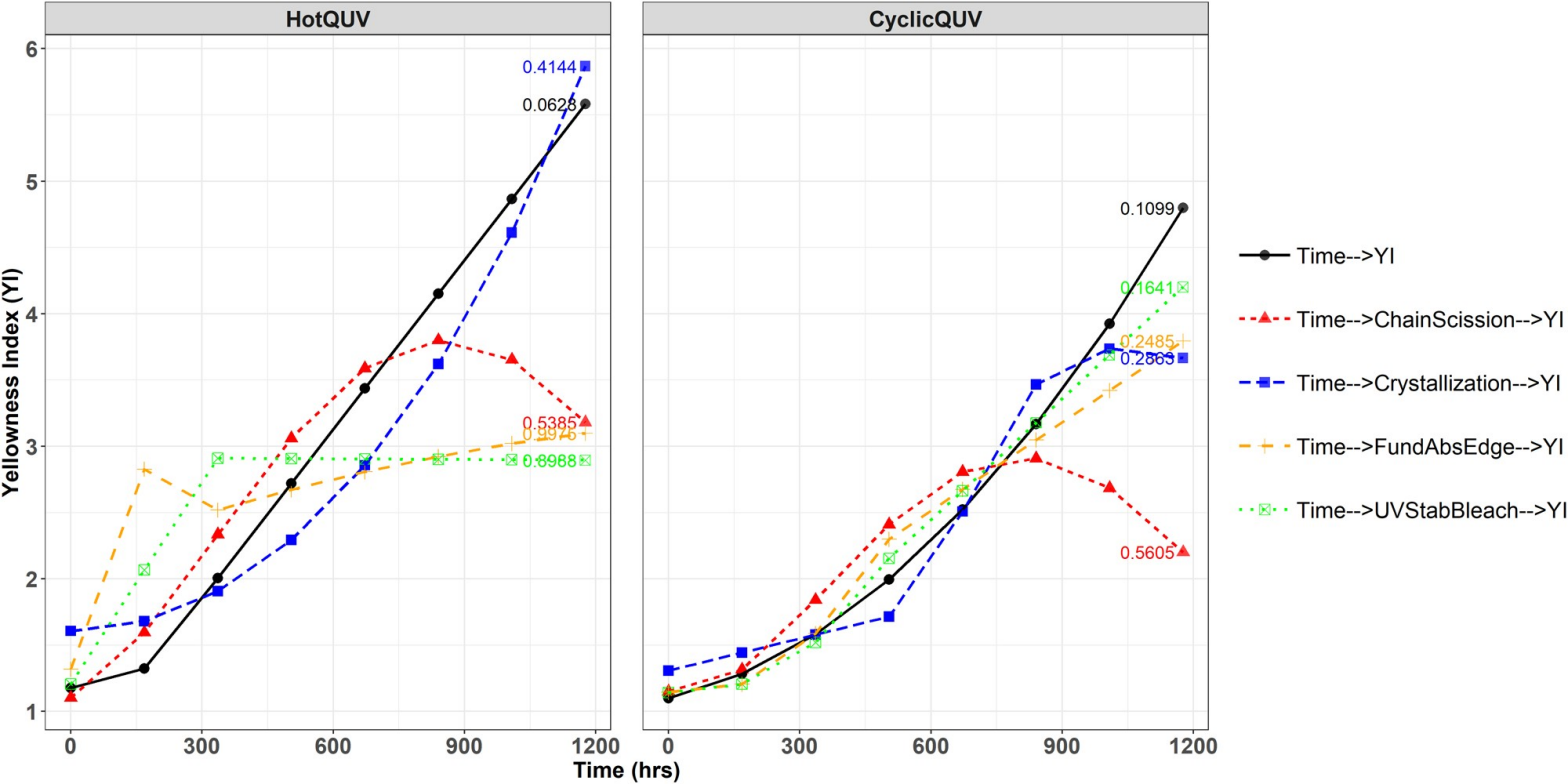
Direct and multi-step (mechanistic) pathway curves for the yellowing of UV stabilized grade. Each exposure step (168 hours) corresponds to one week of exposure and the total exposure time (1176 hours) of seven steps corresponds to seven weeks of exposure. RMSE values are given for each pathway showing the predictive powers of the models.

The mechanistic pathway curves for yellowing under both exposure conditions can be seen in Fig 10. Crystallization in the HotQUV exposure and bleaching of the UV stabilizer in the CyclicQUV exposure closely predicts the direct time to YI path throughout the entire exposure as evident from their small RMSE values. Even though the chain scission variable gives similar YI values within approximately 750 hours under both exposures, it underestimates the YI development afterwards. For the haze formation (see S10 Fig), the mechanistic path through UV stabilizer bleaching depicts almost a perfect match to direct path as expected from its relation to time and haze formation. The paths through crystallization and fundamental absorption edge are also trending well with the haze formation especially within 900 hours of exposure; however, they are poorly predicting the final haze (%) values.

## Discussion

### The effect of exposures on degradation

While UV light exposures caused intense yellowing, the addition of condensed humidity in CyclicQUV also led to strong haze formation. Neither were notable under the heat and humidity exposures with no irradiance content. Detailed discussion of both degradation mechanisms can be found elsewhere [48].

**Fig 11:**
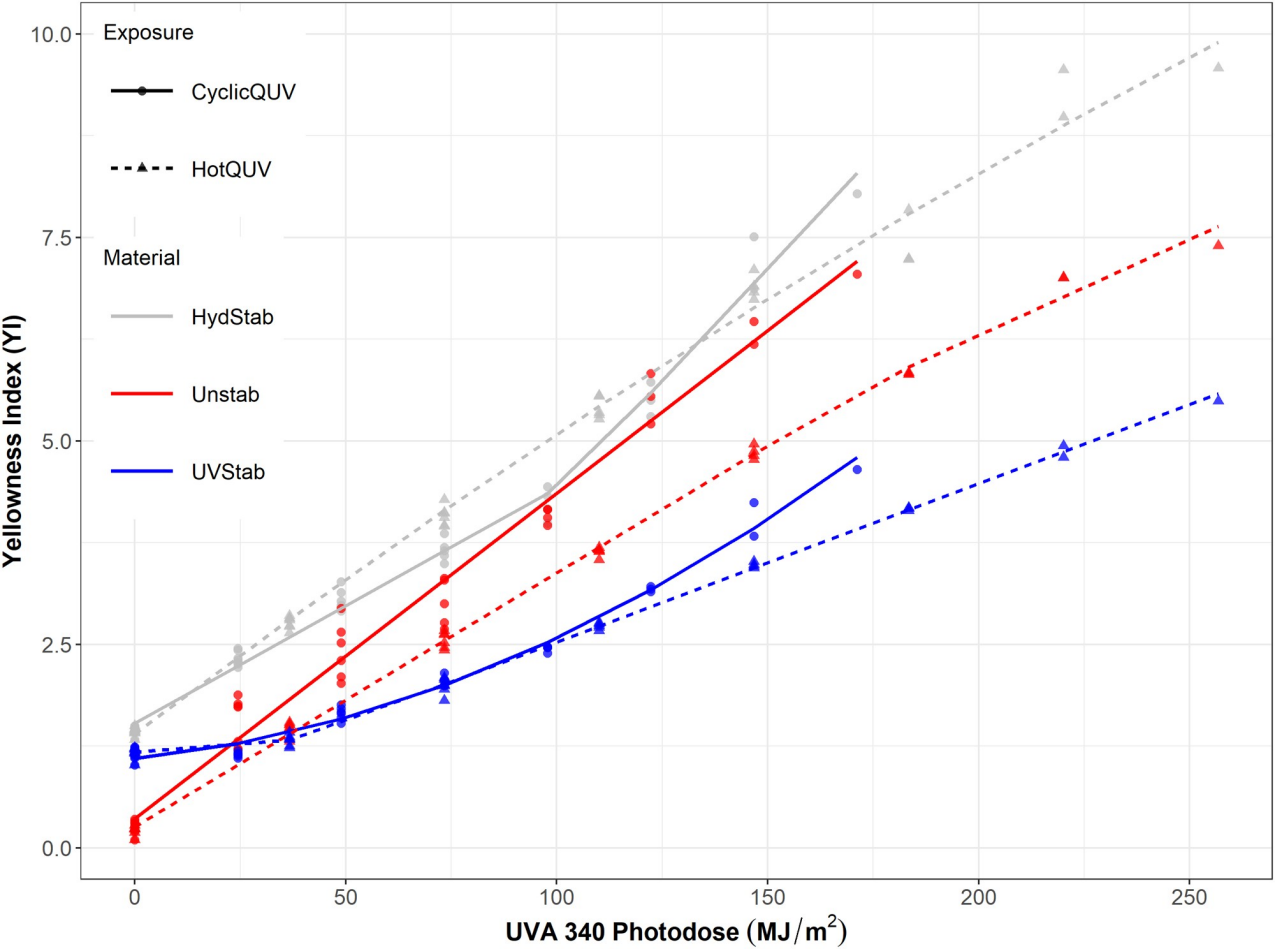
The change in YI as a function of photo-dose.

For the equal amount of applied UVA-340 photo-dose in Fig 11, yellowing in CyclicQUV is greater than that in HotQUV, particularly for the unstabilized grade. This can infer that UV light is not the sole stressor leading to yellowing, but moisture can also contribute to its development. Hydrolysis results in the scission of polymer chains at the ester linkage, leading to the formation of carboxyl and hydroxyl end groups. These acids can also act as catalyst and promote further degradation, i.e., autocatalytic effect. So the formation of reactive species and additional chain scissions as a result of moisture induced reactions, seem to accelerate the formation of yellowing chromophores during UV light exposure.

Haze formation primarily occurs under hydrolytic conditions probably due to partial crystallization. There are two mechanisms by which crystallization occurs during degradation [65]. 1) The extraction of low molecular weight compounds: weight loss due to water soluble small polymer fragments decreases the fraction of amorphous phase, and thus increases crystallinity. 2) Chemi-crystallization process: large number of chain scissions of previously entangled chains in the amorphous phase leads to increased mobility and re-arrangement of chains into crystalline structure. Both mechanisms can be in effect under the CyclicQUV exposure because of the combination of UV light and moisture. Temperature cycling in the CyclicQUV exposure can also cause volumetric changes. This thermal (or even mechanical) expansion or contraction can induce internal stresses and promote cracking or crazing in the bulk or on the surface, leading to further hazing. Solubility and diffusion of water molecules (i.e., plasticization effect) and oxygen in the polymer matrix can also be influenced by the cyclic temperature changing the hydrolysis reaction rates and pathways.

The CyclicQUV exposure with combined stressors generated complex degradation mechanisms: light-induced and moisture-induced reactions produced synergistic effects, leading to some level of acceleration in degradation kinetics. Considering the strong influence of UV light on the degradation of PET and the synergistic and acceleration effects of combined stressors, including UV light in heat and humidity testing should be considered in the standards applied to test the reliability of PV modules. Because of the presence of complex degradation pathways due to the multi-factor stress conditions in real-world outdoor exposures, this approach would allow more accurate understanding of degradation mechanisms and lifetime predictions of the PV systems and components.

### The effect of chemical composition on degradation

The effect and presence of stabilization strategies against the UV light or hydrolysis was noticeable from the occurrence of change points (i.e., onset of yellowing and haze formation). During the induction period stabilization protected the polymers from the damaging stressors and no significant changes were observed. However, change points were observed when the stabilization was lost and hence the degradation mechanisms accelerated once the stabilizing additive was consumed. The type and strategy of the stabilization play a critical role as discussed in S1 Appendix.

Aside from the stabilizer additives, initial diethylene terephthalate (DEG) content (see S1 Appendix), crystallinity (see S2 Appendix), intrinsic viscosity and molecular weight (see S3 Appendix), and carboxyl end group (CEG) content (see S4 Appendix), are known to have major impacts on PET’s stability against UV and hydrolytic degradation [64, 66]. The hydrolytically stabilized grade has a low initial CEG content, so as to have enhanced hydrolytic stability, yet it also has a high DEG content, low crystallinity, and high intrinsic viscosity. These, in fact, can promote hydrolytic attack and discoloration. On the other hand, the UV stabilized grade has a high CEG and a low DEG content and these can inversely impact its UV stability. Upon exposure, as reported in S4 Appendix, in terms of the change in CEG content under both light and humid conditions, while a drastic increase was observed in the hydrolytically stabilized, a moderate increase was observed in the UV stabilized grade probably due to the presence of UV stabilizer.

The hydrolytically stabilized grade was observed to be slightly more stable than the other two grades under hydrolytic conditions, probably due to the end-capped and thus decreased amount of carboxyl end groups (CEGs) in the initial polymers, but this stabilization strategy was not very effective. Similarly, the bleaching of the UV stabilizer additive in the UV stabilized grade in a short time frame left the polymer susceptible to UV light and led to drastic yellowing. So for both stabilized grades, stabilization provided protection to a some degree under the applied accelerated exposure conditions; however, further studies are required under the real-world conditions to determine if these grades are suitable for PV applications considering the expected lifetime warranty of 25 years.

### Development of degradation pathway models

For the development of YI, more variables became strongly correlated in the CyclicQUV exposure when compared to the HotQUV exposure. This suggests the effect of multiple stressors and cyclic exposure condition leading to different degradation mechanisms acting simultaneously. Even though some multi-step mechanistic paths successfully predicted the direct paths, deviations were observed especially after 750 hours of exposure. This behavior can be related to the univariate nature of the analysis: interactions between mechanistic variables are not explicitly considered to be acting on the final response and their presence might be altering the overall pathway. Note that all of the singular paths either carry either a quadratic functional form or a change point model so that the resulting mechanistic paths might be affected at the extent of exposure as they were obtained by substituting one equation into another. Another factor could be the cyclic nature of the CyclicQUV exposure: light and moisture can cause complex interactions within the material specifically on the chemical mechanisms. These complex mechanisms might also arise from the autocatalytic nature of the hydrolysis, during which the formation of active carboxyl end groups can lead to subsequent reactions, particularly in the CyclicQUV exposure.

NetSEM modeling utilizes univariate relationships and is able to capture change points seen in the response variables [60]. Change points (i.e., induction periods or delayed onset of degradation) usually consists of two different mechanisms (i.e., two different functional forms, controlling the overall behavior). For instance, in the case of yellowing of the UV stabilizer under the light exposures, two linear relationships were observed: retarded yellowing due to the effect of UV stabilizer and a drastic increase in yellowing after the UV stabilizer bleached. Similarly, the haze formation was seen in two functional forms: a delayed onset of hazing and a substantial increase after the damage accumulation. Capturing these differing and complex mechanisms due to change points enhanced the significance of the pathway models. Additive relationships between mechanistic variables will be included in this modeling approach to further improve the power of this analysis.

Moreover, in real-world, multiple mechanisms can be active and their synergistic interactions can make for a complex network of degradation pathways that are impacted by multiple stressors, their quantities (or levels), and the resulting rates of diffusion and reactions. So to address the complex nature of this problem, the development of statistical and predictive models including their dependence on both stressors and responses using multi-variate data science and analytics methods is essential.

## Conclusion

Degradation of three PET grades was performed under different accelerated weathering exposures and the changes in polymers were evaluated by various spectroscopic techniques. The UV stabilized grade was found to be slightly more stable than the other grades; however, the bleaching of the stabilizer was evident after a short exposure time. Lesser yellowing in cyclic exposure was first attributed to the smaller photo-dose content; however, the effect of moisture on yellowing was evident on a photo-dose basis. No haze formation was observed in the UV light exposure despite the high level of yellowing, but it developed markedly under the cyclic conditions due to the presence of cyclic light, heat, and humidity. The stabilization involved in these grades were found to be effective only for short time frames under the applied accelerated conditions, but left the materials vulnerable to degradation afterwards. Real-world studies are therefore needed to elucidate the effectiveness of these grades for applications such as photovoltaic modules which require long lifetime performance.

Measured responses were used in the netSEM analysis and degradation pathway diagrams were generated. Due to the cyclic conditions, more mechanistic variables were found to be acting on the performance level response when compared to constant stress conditions. Time dependent equations that correspond to these degradation pathways were also derived and contributions from mechanistic variables were determined. The pathway models were successfully projected by the contributions from mechanistic variables; however, complex interactions between degradation mechanisms lead to some deviations in the prediction.

## Supporting information

S1 Appendix Chemical composition and catalyst metal trace analysis. (PDF)

S2 Appendix Crystallinity measurement via Differential Scanning Calorimetry (DSC). (PDF)

S3 Appendix Intrinsic viscosity (IV) and molecular weight (M_w_) measurements. (PDF)

S4 Appendix Carboxyl end group (CEG) content analysis. (PDF)

S1 Fig netSEM diagram for the hazing of UV stabilized grade under the CyclicQUV exposure. (TIFF)

S2 Fig netSEM diagram for the yellowing of unstabilized grade under the HotQUV exposure. (TIFF)

S3 Fig netSEM diagram for the yellowing of unstabilized grade under the CyclicQUV exposure. (TIFF)

S4 Fig netSEM diagram for the hazing of unstabilized grade under the CyclicQUV exposure. (TIFF)

S5 Fig netSEM diagram for the yellowing of hydrolytically stabilized grade under the HotQUV exposure. (TIFF)

S6 Fig netSEM diagram for the yellowing of hydrolytically stabilized grade under the CyclicQUV exposure. (TIFF)

S7 Fig netSEM diagram for the hazing of hydrolytically stabilized under the CyclicQUV exposure. (TIFF)

S8 Fig Direct and multi-step (mechanistic) pathway curves for the yellowing of unstabilized grade. (TIFF)

S9 Fig Direct and multi-step (mechanistic) pathway curves for the yellowing of hydrolytically stabilized grade. (TIFF)

S10 Fig Direct and multi-step (mechanistic) pathway curves for the hazing of the three PET grades. (TIFF)
