# Supplementary material for: Temporal evolution and pathway models of poly(ethylene-terephthalate) degradation under multi-factor accelerated weathering exposures
Source: PLoS One. 2019 Feb 15;14(2):e0212258. doi: 10.1371/journal.pone.0212258 (PMC6377120; doi:10.1371/journal.pone.0212258)
Supplement: S1 Appendix — (PDF) [file pone.0212258.s001.pdf]

S1 Appendix. Chemical Composition and Catalyst Metal Trace Analysis

The PET grades used in this research were selected from commercial products, two of which were advertised as having stabilization against UV and hydrolytic degradation. However, as discussed in the study, both type of stabilization strategies were not found to be efficient under the applied exposure conditions.

For a detailed understanding of the materials' chemical compositions, Nuclear Magnetic Resonance Spectroscopy (NMR) analysis of the three PET grades was performed and provided by 3M as part of our collaboration. For qualification of catalyst content of the three PET grades, Inductively Coupled Plasma Mass Spectroscopy (ICP-MS) and Energy Dispersive X-ray (EDX) techniques were also conducted and provided by Auriga Polymers in order to determine the starting synthesis material, i.e., DMT (dimethyl terephthalate) or TPA (terephthalic acid) based PET synthesis. These techniques allow elemental analysis with detection limits in the ppm levels. It is TPA based synthesis if only antimony (Sb) is used as a catalyst and it is DMT based synthesis if other metals such as copper (Cu), manganese (Mn), magnesium (Mg), cobalt (Co), zinc (Zn), etc., are present in addition to antimony (Sb). It is to be noted that these analyses were acquired on a single sample from each grade and therefore should not be taken as a statistically representative sampling of a commercial product production.

According to the NMR analysis results, the three grades contains standard PET resonances with no co-monomers. The formation of side products and end groups that is known to occur during esterification and polycondensation reactions was also detected. It was found that there was no evidence of any additive in the hydrolytically stabilized grade. For the UV stabilized grade, a compound with a chemical formula of $C_{22}H_{12}N_2O_4$ (a benzoxazinone type UV stabilizer commercialized under the name of Cyasorb UV 3638 by CYTEC [1] was identified as the UV stabilizer. Pickett [2] reports that this type of UV stabilizer was found to have a poor photo-stability that limits their use in polyesters. This might well explain the rapid deterioration seen in the UV stabilized grade. The intended protection of the UV stabilizer was found to be in effect only during the first exposure step. Full chemical compositions of the three grades can be found in Table S1.1.

Table S1.1. Chemical compositions of the three PET grades. Note that all values are in weight %. TP: terephthalate, EG: ethylene glycol, DEG: diethylene glycol, TEG: triethylene glycol, Veg: vinyl end group, MEeg: methyl ester end group.

Grade	TP	EG	DEG	TEG	Veg	MEeg	UV Stabilizer
Hyd. stabilized	68.1	30.4	1.46	0.03	0.01	0.02	-
Unstabilized	68.3	30.8	0.85	0.01	0.02	-	-
UV stabilized	67.4	30.5	0.56	0.01	0.02	0.03	1.5

When comparing the chemical compositions of these polymers, the hydrolytically stabilized grade seems to be almost identical to the unstabilized grade, with the diethylene glycol (DEG) and triethylene glycol (TEG) content being the major difference. Although there was no stabilizer additive found, it can be suggested that hydrolytic stabilization was achieved by deactivating reactive polymer chain ends, i.e., carboxyl end groups, by an end-capping technique. This could provide hydrolysis resistance above standard PET; however, the presence of large amounts of DEG and TEG side products might affect hydrolytic stability adversely as they being preferential sites for hydrolytic attack. Moreover, whereas the presence of the methyl ester end group indicates that the polymer synthesis was conducted by trans-esterification reaction (DMT based synthesis), the presence of large amounts of DEG and TEG side products suggests the direct esterification reaction (TPA based synthesis). Therefore, analysis of catalyst metal trace and carboxyl end group (CEG) content analyses become

necessary to elucidate the mechanism of hydrolytic stability and synthesis route. Other than the presence of a UV stabilizer, the UV stabilized grade has also a similar composition to the unstabilized grade. The major differences are the presence of methyl ester end groups and lesser amount of DEG side product than the other two grades, both of which suggest trans-esterification reaction (DMT based) as the synthesis route. The lesser amount of DEG content might also provide stability above unstabilized PET as DEG groups play a role in forming discoloring chromophores.

Further analyses via Inductively Coupled Plasma Mass spectrometry (ICP-MS) that can characterize metal traces in the bulk polymers were performed for more accurate determination of catalyst content and thus starting material. Table S1.2 shows the ICP-MS analysis performed for metals typically found in PET polymers. Note that given values represent the ppm levels of metal traces. The presence of a large amount of manganese (Mn), in addition to Sb, suggests that all three grades were synthesized from DMT rather than TPA based. The presence of phosphorus (P) in moderate ppm levels is probably because it is added at the end of esterification process to arrest the catalytic activity. The ppm levels for Ti and Ge were found to be negligible. Only the UV stabilized grade seems to have Co, in addition to Mn, as a polycondensation catalyst.

Table S1.2. ICP-MS metal trace analysis of the three PET grades in ppm levels

	**Hydrolytically stabilized**	**Unstabilized**	**UV stabilized**
**Sb**	186.50 ± 5.37	174.45 ± 0.64	294.00 ± 3.68
**Mn**	61.20 ± 2.49	80.50 ± 0.31	82.00 ± 1.36
**Co**	< 1	9.51 ± 0.44	< 1
**P**	54.77 ± 1.21	79.70 ± 1.36	53.08 ± 1.42
**Ti**	< 1	<1	< 1
**Ge**	< 1	<1	< 1

In addition, the organic components of the films were burned with a flame and the ash residue was examined by Energy Dispersive X-ray (EDX). Scans of the three grades (not included here) show qualitative displays of all elements present in the samples. The presence of Sb, Mn, and P was clearly seen, confirming the previous observations. The presence of Si was attributed to the use of slip agent. Small amounts of Na and Al were also present in insignificant quantities and were probably contaminates from the analysis process. Overall, in the light of these observations, it can be inferred that the all three grades used in this work were synthesized via DMT based trans-esterification route.

References

1. Cytec. Cyasorb UV-3638F. Available from: `https://www.cytec.com/products/cyasorb-uv-3638f`.

2. Pickett JE. Permanence of UV Absorbers in Plastics and Coatings. In: Hamid SH, editor. Handbook of polymer degradation. 2nd ed. No. 21 in Environmental science and pollution control series. New York: Marcel Dekker; 2000. p. 163 – 190.
