# Supplementary material for: Temporal evolution and pathway models of poly(ethylene-terephthalate) degradation under multi-factor accelerated weathering exposures
Source: PLoS One. 2019 Feb 15;14(2):e0212258. doi: 10.1371/journal.pone.0212258 (PMC6377120; doi:10.1371/journal.pone.0212258)
Supplement: S2 Appendix — (PDF) [file pone.0212258.s002.pdf]

S2 Appendix. Crystallinity Measurement via Differential Scanning Calorimetry (DSC)

The differential scanning calorimetry (DSC) measurements were performed on the unexposed baseline and very last exposed samples under the HotQUV and CyclicQUV exposures to determine changes in crystallinity before and after degradation. The method selected follows three cycles: heating of the sample from 25 to 300 °C (first heating cycle), subsequent cooling from 300 to 25 °C (cooling cycle), and heating up to 300 °C (second heating cycle). The heating and cooling rate were 10 °C/min and the measurements were conducted under a flow of dry nitrogen (50 ml/min) for all cycles. From the first heating cycle, the percent crystallinity was then calculated using Eq S2.1.

$$\%\text{Crystallinity} = \frac{\Delta H_m - \Delta H_{cc}}{\Delta H_m^0} \times 100 \tag{S2.1}$$

where the melting enthalpy of the perfect crystal (ΔH_m^0) was taken as 140 J/g [1].

The first heating cycle was conducted in order to remove the thermal and stress history of the samples to obtain on the extent of crystallinity in the degraded samples. Because the aging induced crystallinity is the point of interest, % crystallinity values were calculated by integrating the area under the melting endotherms from the first heating cycles. Note that glass and cold crystallization transitions could not be resolved clearly in the curves and therefore no glass transition or cold crystallization temperatures (T_g) were reported. Overall, these results will allow a direct measure of the change in crystallinity, which are used to confirm the indirect results obtained through IR absorption of the *trans* ethylene glycol unit, as conformational IR bands are affected by orientation and heat treatment.

Table S2.1 gives the thermal transitions of the degraded samples during the first heating cycle. From the baseline measurements, it can be seen that the three grades have similar crystallinity contents. Upon exposure, changes in melting characteristics from baseline to exposed samples are clear in all grades: reduction in melting points and increase in % crystallinity. The HotQUV exposure seems to cause more reduction in melting points than the CyclicQUV exposure, but the difference is relatively small. There is only a slight change in the UV stabilized grade compared to the other two grades, which can be attributed to its stability against UV light. Due to large uncertainties for the calculated % crystallinity values, a direct comparison between exposures can not be made; however, aging induced crystallinity is obvious when compared to the unexposed baseline values. It should be noted that when compared the baseline values of the three grades, the hydrolytically stabilized grade has the lowest initial crystallinity. Due to the impermeable nature of the crystalline phase, more crystalline content in this grade would have been expected for better hydrolytic stability.

There are two features that should be noticed in the DSC curves (not shown here): the shift of the melting point toward lower temperatures and the broadening of the melting peak. One possible explanation for these is the aging-induced newly formed crystallites that are smaller in size with a lower degree of perfection compared to the initial crystallites in the unexposed baseline samples, resulting in a broader range of melting temperatures and a broader melting endotherm. Larger broadening in the first heating cycle compared to that in the second heating cycle also supports this phenomenon as slow heating in the second heating cycle followed by the slow cooling cycle helped form larger crystallites with a higher degree of perfection, that displayed narrower range of melting temperatures and more sharper melting endotherms in the DSC curves. There is another interpretation of this broadening in the literature [2, 3] such that initial crystallites breaks apart into smaller crystallites that have a lower melting temperatures than the larger crystallites; however, this seems to be valid for conditions under physical aging processes.

Table S2.1. DSC thermal transitions in PET samples degraded under the HotQUV and CyclicQUV exposures - First heating cycle. $\mathbf{T_m}$ is the melting point and $\mathbf{\Delta H_m}$ is the enthalpy of melting.

**Grade - Exposure**	$\mathbf{T_m}$ (°C)	$\mathbf{\Delta H_m}$ **(J/g)**	**% Crystallinity**
Hyd. stabilized - Baseline	254.52 ± 0.54	38.92 ± 3.73	27.80 ± 2.66
Hyd. stabilized - HotQUV	246.67 ± 1.93	56.33 ± 5.32	40.24 ± 3.80
Hyd. stabilized - CyclicQUV	250.49 ± 0.73	57.70 ± 6.90	41.22 ± 4.93
Unstabilized - Baseline	257.59 ± 0.19	39.77 ± 8.87	28.40 ± 6.34
Unstabilized - HotQUV	245.30 ± 0.89	58.54 ± 4.70	41.81 ± 3.36
Unstabilized - CyclicQUV	248.33 ± 1.30	60.05 ± 3.20	42.89 ± 2.28
UV stabilized - Baseline	258.22 ± 0.67	43.64 ± 11.58	31.17 ± 8.27
UV stabilized - HotQUV	256.21 ± 0.66	61.16 ± 6.33	43.68 ± 4.52
UV stabilized - CyclicQUV	256.28 ± 1.65	48.87 ± 13.73	34.90 ± 9.81

References

1. Wunderlich B. Equilibrium melting of flexible linear macromolecules. Polymer Engineering & Science. 1978;18(6):431–436. doi:10.1002/pen.760180603.

2. Arrieta C, Dong Y, Lan A, Vu-Khanh T. Outdoor weathering of polyamide and polyester ropes used in fall arrest equipment. Journal of Applied Polymer Science. 2013;doi:10.1002/app.39524.

3. Quintanilla L, Rodríguez-Cabello JC, Jawhari T, Pastor JM. Structural analysis of injection-moulded semicrystalline polymers by Fourier transform infra-red spectroscopy with photoacoustic detection and differential scanning calorimetry: 1. Poly(ethylene terephthalate). Polymer. 1993;34(18):3787–3795. doi:10.1016/0032-3861(93)90502-2.
