# Supplementary material for: Temporal evolution and pathway models of poly(ethylene-terephthalate) degradation under multi-factor accelerated weathering exposures
Source: PLoS One. 2019 Feb 15;14(2):e0212258. doi: 10.1371/journal.pone.0212258 (PMC6377120; doi:10.1371/journal.pone.0212258)
Supplement: S3 Appendix — (PDF) [file pone.0212258.s003.pdf]

S3 Appendix. Intrinsic Viscosity (IV) and Molecular Weight (M_w) Measurements

Because the change in molecular weight is a critical parameter in determining degradation pathways, starting and ending molecular weights were calculated from intrinsic viscosity (IV) measurements. The test method is defined in ASTM D4603-03 standard [1]. The relationship between intrinsic viscosity and molecular weight is shown in Eq S3.1 as described in Allen et al. [2].

$$\eta = 1.7 \times 10^{-4}[\overline{M}_n]^{0.83} = 1.6 \times 10^{-4}[\overline{M}_w]^{0.76} \tag{S3.1}$$

Theoretical calculations of number of total end groups and the number of chain scissions can also be done by using Eq S3.2 and Eq S3.3, respectively.

$$\text{Total end groups} = \frac{57.46}{\eta^{1.205}} \tag{S3.2}$$

$$\text{Chain scissions per molecule} = \frac{\overline{M}_{n0}}{\overline{M}_{nt}} - 1 \tag{S3.3}$$

where $\overline{M}_{n0}$ and $\overline{M}_{nt}$ are the number average molecular weights at time zero and time t.

Table S3.1 shows the measured intrinsic viscosity (η) and calculated number average molecular weight $\overline{M}_n$ values for each grade under the HotQUV and CyclicQUV exposures. There are, however, some points that should be noted about the reported values. 1) Samples that were exposed at differing times were combined in order to have the required amount of material for testing and therefore no comparison between grades and exposures as a function of time can be made except for the unexposed baseline samples. This will only allow an overall comparison of changes for before and after degradation. 2) The hydrolytically stabilized and unstabilized grades exposed under HotQUV exposure did not dissolve completely, so measured intrinsic viscosity values for these particular grade-exposure couples might be slightly smaller than the actual values. 3) The measurements were conducted only once for each grade-exposure couple due to limited amount of materials and therefore should not be taken as a statistically representative of measurements.

When comparing the unexposed baseline samples, the hydrolytically stabilized grade has the highest and the unstabilized and UV stabilized grades have similar intrinsic viscosity and thus molecular weight values. Upon degradation, a reduction in molecular weight is clear for all grades and exposure types. Similarly, there is a visible increase in the formed total end groups and the number of chain scissions per molecule. Except for the unstabilized grade, the CyclicQUV exposure seems to cause more degradation than the HotQUV exposure; however, a direct comparison can unfortunately not be made, as stated before. In general, it is evident that both exposures have caused a pronounced decrease in η and $\overline{M}_n$ and increase in the number of total end groups as a result of photodegradation and hydrolysis induced chain scissions. The presence of undissolved materials in the unstabilized and hydrolytically stabilized grades exposed under the HotQUV exposure suggest the formation of cross-links, a high molecular weight compounds, that play role in discoloration and brittleness.

Table S3.1. Intrinsic viscosity, molecular weight, number of total end groups, and number of chain scissions of the three PET grades before and after degradation. IV (η) is intrinsic viscosity (dL/g) and $\overline{\mathrm{M}}_{\mathrm{n}}$ is number average molecular weight. Note that the samples with * had very small amounts of materials that went undissolved.

**Grade - Exposure**	**IV(η)**	$\overline{\mathrm{M}}_{\mathrm{n}}$	**Total end groups**	**Chain scissions**
Hyd. stabilized - Baseline	0.648	20,635	97	0
Hyd. stabilized - HotQUV*	0.593	18,544	108	0.11
Hyd. stabilized - CyclicQUV	0.495	14,917	135	0.38
Unstabilized - Baseline	0.564	17,457	115	0
Unstabilized - HotQUV*	0.375	10,676	188	0.64
Unstabilized - CyclicQUV	0.457	13,548	148	0.29
UV stabilized - Baseline	0.573	17,793	114	0
UV stabilized - HotQUV	0.503	15,208	132	0.17
UV stabilized - CyclicQUV	0.497	14,990	136	0.19

References

1. ASTM D4603-03(2011)e1, Standard Test Method for Determining Inherent Viscosity of Poly(Ethylene Terephthalate) (PET) by Glass Capillary Viscometer. West Conshohocken, PA: ASTM International; 2011. Available from: www.astm.org.

2. Allen NS, Edge M, Mohammadian M, Jones K. Physicochemical aspects of the environmental degradation of poly(ethylene terephthalate). Polymer Degradation and Stability. 1994;43(2):229–237. doi:10.1016/0141-3910(94)90074-4.
