# Supplementary material for: Temporal evolution and pathway models of poly(ethylene-terephthalate) degradation under multi-factor accelerated weathering exposures
Source: PLoS One. 2019 Feb 15;14(2):e0212258. doi: 10.1371/journal.pone.0212258 (PMC6377120; doi:10.1371/journal.pone.0212258)
Supplement: S4 Appendix — (PDF) [file pone.0212258.s004.pdf]

S4 Appendix. Carboxyl End Group (CEG) Content Analysis

The carboxyl end group concentration in PET plays a major role in hydrolytic stabilization due to their autocatalytic effect in hydrolysis reactions. Therefore, determining initial and final CEG content in the PET films will help assess useful information about the hydrolytic stability during aging, specifically for the hydrolytically stabilized grade. The CEG content analysis has been performed via titration of the carboxyl end groups as defined by ASTM D7409-15 [1] standard.

Table S4.1 provides the initial and final carboxyl end group concentrations of the degraded polymers under the HotQUV and CyclicQUV exposures. The same points reported for intrinsic viscosity measurements should also be taken into account for these CEG measurements. Because initial and final CEG concentration have an utmost importance in determining reaction routes and hydrolytic stability, measured concentrations of CEGs will help elucidate some of the issues addressed before and will confirm the validity of the use of IR band in the netSEM analysis as a mechanistic variable for chain scission behavior.

When comparing unexposed baseline samples, the hydrolytically stabilized has the lowest CEG content among other grades despite its very high molecular weight. However, a significant increase in CEG content is evident after exposure: 9.5-fold increase in HotQUV and 8.2-fold increase in CyclicQUV. Since measurements were taken on samples exposed for similar durations under both exposure conditions (fifth and sixth steps), a rough comparison can be made such that the HotQUV exposure seems to lead to more CEG formation than the CyclicQUV exposure. This suggest that the hydrolytic stability of this particular grade has been achieved via aforementioned end-capping technique with which active carboxylic acid end groups were deactivated.

The unstabilized grade has a slightly higher CEG content in the unexposed baseline sample than the hydrolytically stabilized grade. Similarly, a marked increase in CEG content is observed under both exposures: 7.6-fold increase in HotQUV and 7-fold increase in CyclicQUV. Considering that the measurements were taken on samples that were exposed for similar durations (fourth and fifth steps) under both exposures, two observations could be made: 1) both exposure conditions have yielded similar CEG contents and 2) when compared the magnitude of increase and exposure times the samples were faced, the unstabilized and the hydrolytically stabilized grades behaved somewhat similarly.

Even though the UV stabilized grade has the highest initial CEG concentration, the increase after the degradation is small compared to the other two grades: 3.6-fold increase in HotQUV and 2.8-fold increase in CyclicQUV. Given that the measurements were conducted on samples that were retained at fourth and fifth exposure steps for both exposure conditions, similar to the unstabilized grade, it can be inferred that the UV stabilized grade is more stable than the other two grades and the HotQUV exposure has led to more CEG formation than the CyclicQUV exposure.

References

1. ASTM D7409-15, Standard Test Method for Carboxyl End Group Content of Polyethylene Terephthalate (PET) Yarns. West Conshohocken, PA: ASTM International; 2015. Available from: `www.astm.org`.

Table S4.1. Carboxyl end group (CEG) concentrations of the three PET grades before and after degradation. Note that the samples with * had very small amounts of materials that went undissolved. KOH volume for blank solution is 0.35 ml.

**Grade - Exposure**	**KOH (ml)**	**CEG (mmol/Kg)**	**Ave. CEG (mmol/Kg)**
Hyd. stabilized - Baseline	1.30<br>1.40	19.3<br>21.0	20.2±1.21
Hyd. stabilized - HotQUV*	9.85<br>9.80	192.6<br>192.5	192.6±0.05
Hyd. stabilized - CyclicQUV	8.55<br>8.65	166.7<br>165.3	166.0±0.94
Unstabilized - Baseline	1.70<br>1.75	27.6<br>28.5	28±0.69
Unstabilized - HotQUV*	10.75<br>10.85	214.8<br>211.4	213.1±2.43
Unstabilized - CyclicQUV	10.15<br>10.05	198.6<br>198.6	198.6±0.01
UV stabilized - Baseline	2.35<br>2.40	40.2<br>41.7	40.9±1.03
UV stabilized - HotQUV	7.50	146.6	146.6
UV stabilized - CyclicQUV	6.00	114.1	114.1
