# Supplementary figures and images for: Temporal evolution and pathway models of poly(ethylene-terephthalate) degradation under multi-factor accelerated weathering exposures
Source: PLoS One. 2019 Feb 15;14(2):e0212258. doi: 10.1371/journal.pone.0212258 (PMC6377120; doi:10.1371/journal.pone.0212258)

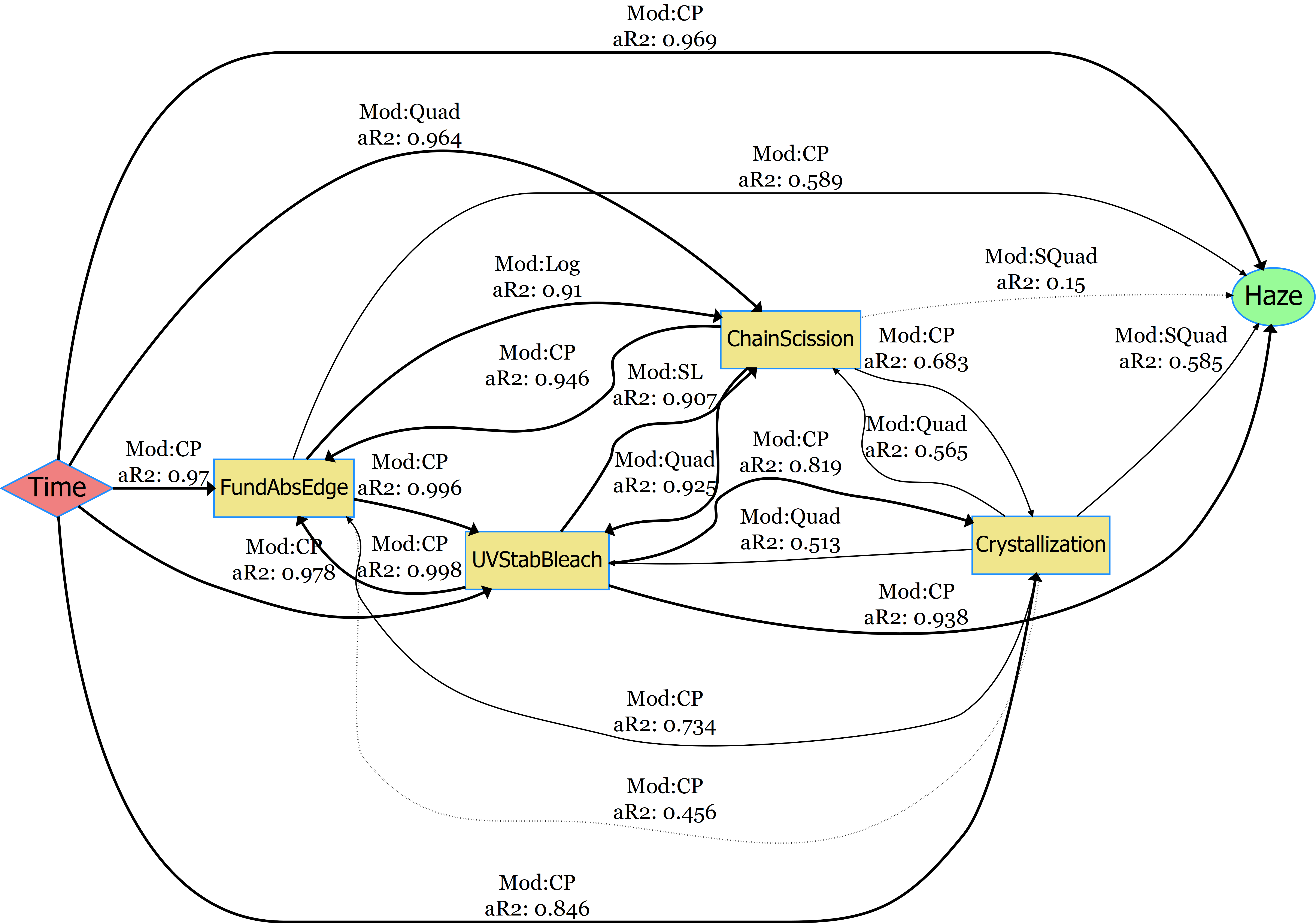

Supplement: S1 Fig — (TIFF) [file pone.0212258.s005.tiff]

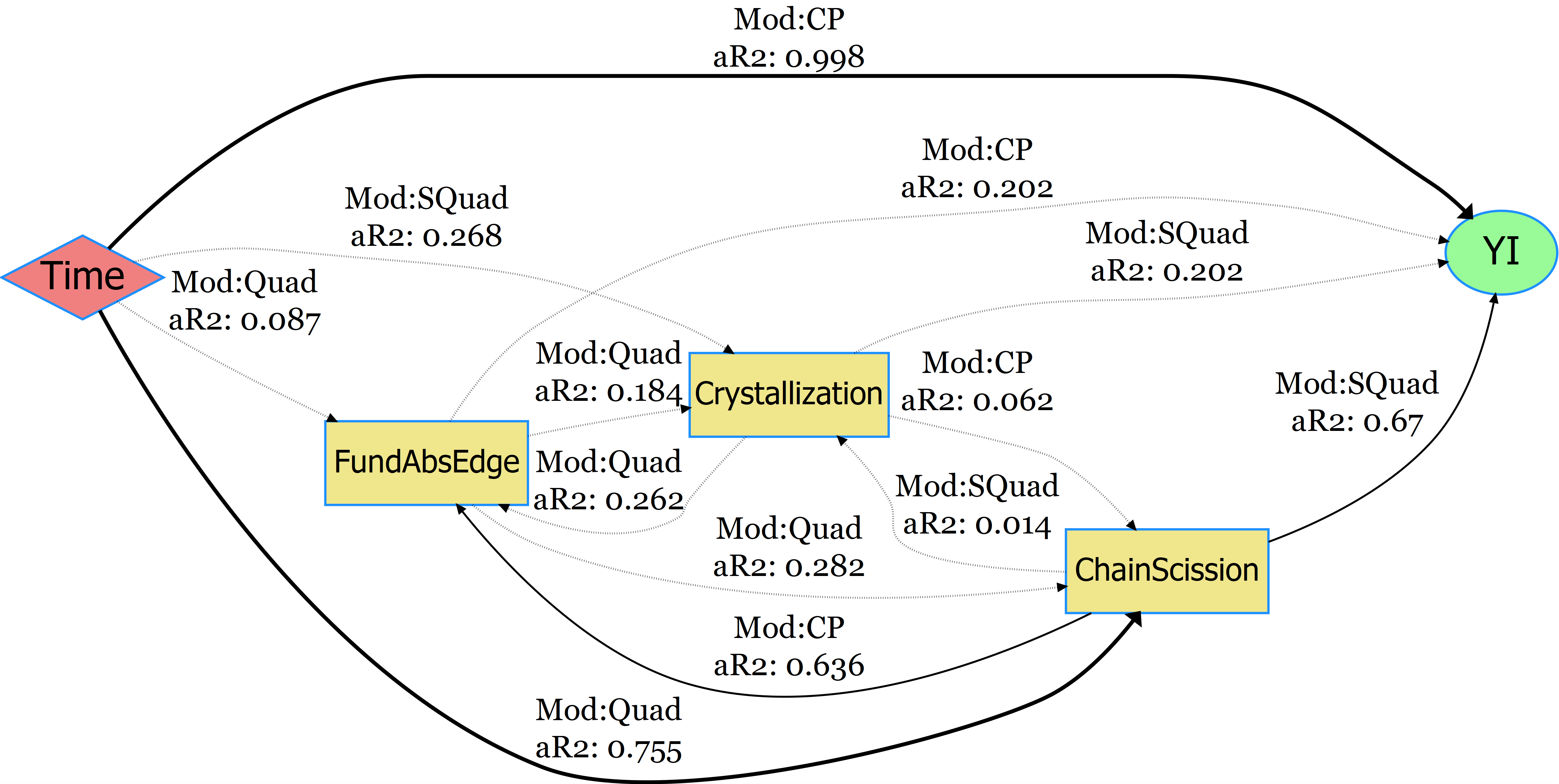

Supplement: S2 Fig — (TIFF) [file pone.0212258.s006.tiff]

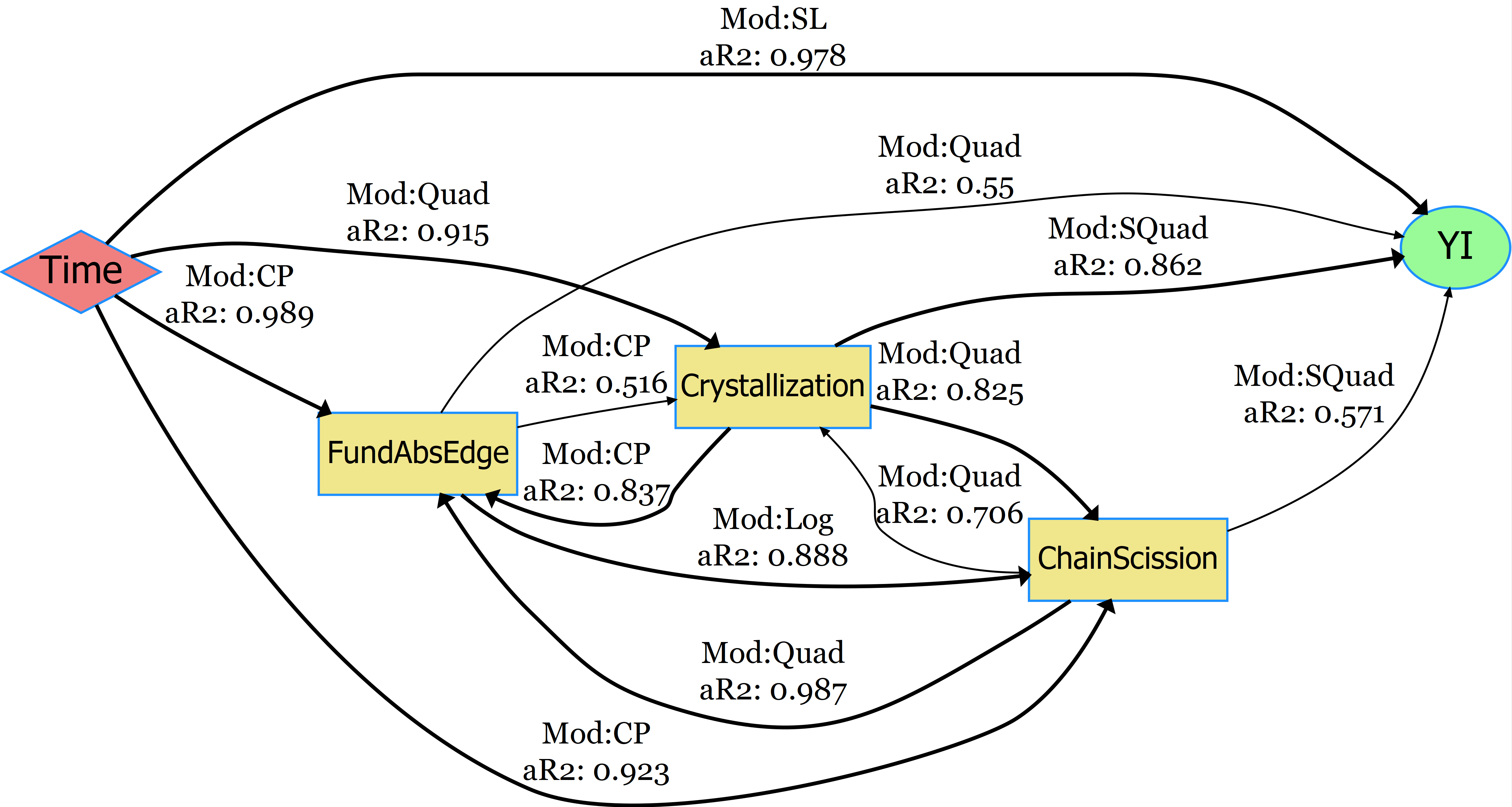

Supplement: S3 Fig — (TIFF) [file pone.0212258.s007.tiff]

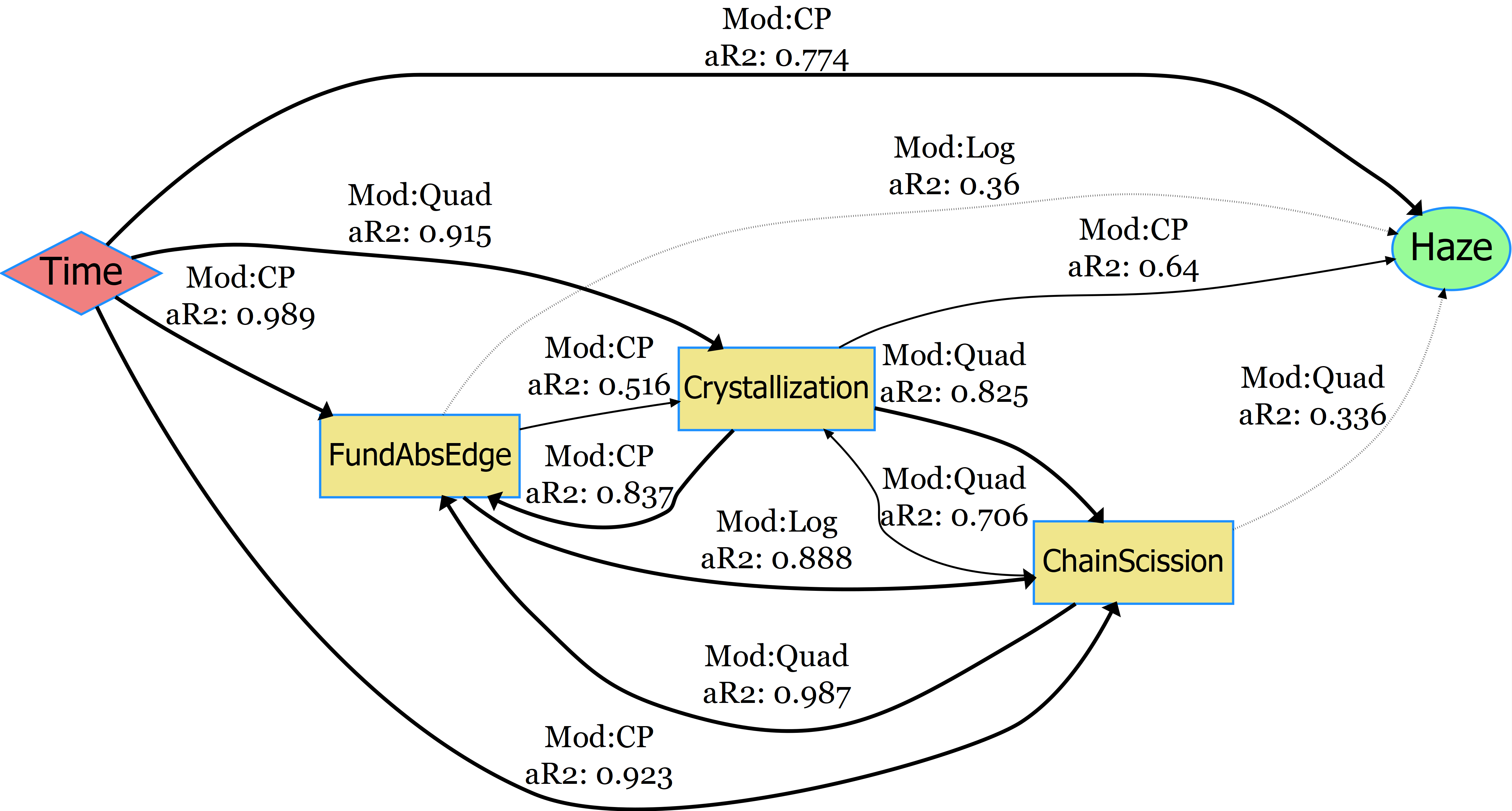

Supplement: S4 Fig — (TIFF) [file pone.0212258.s008.tiff]

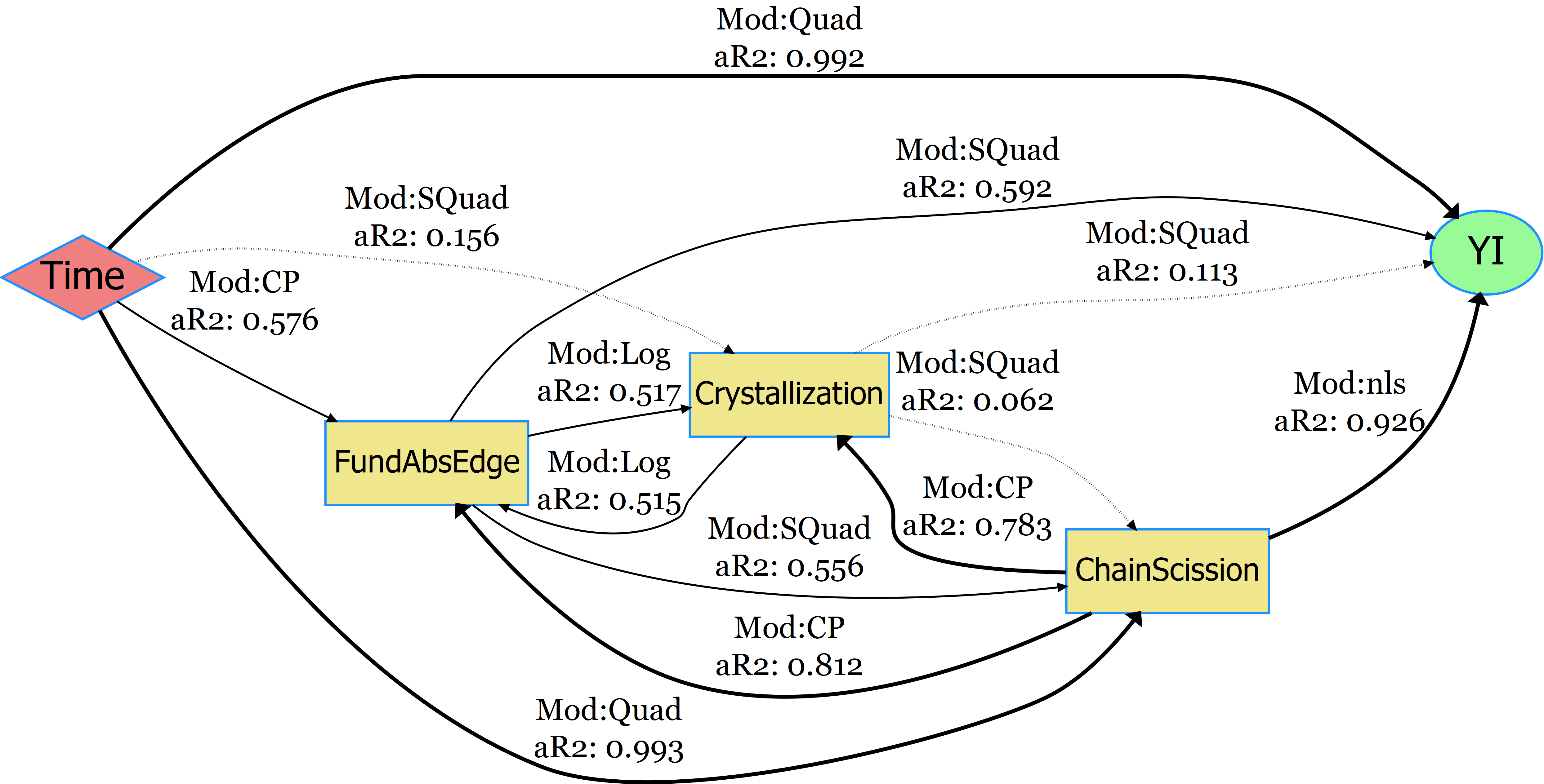

Supplement: S5 Fig — (TIFF) [file pone.0212258.s009.tiff]

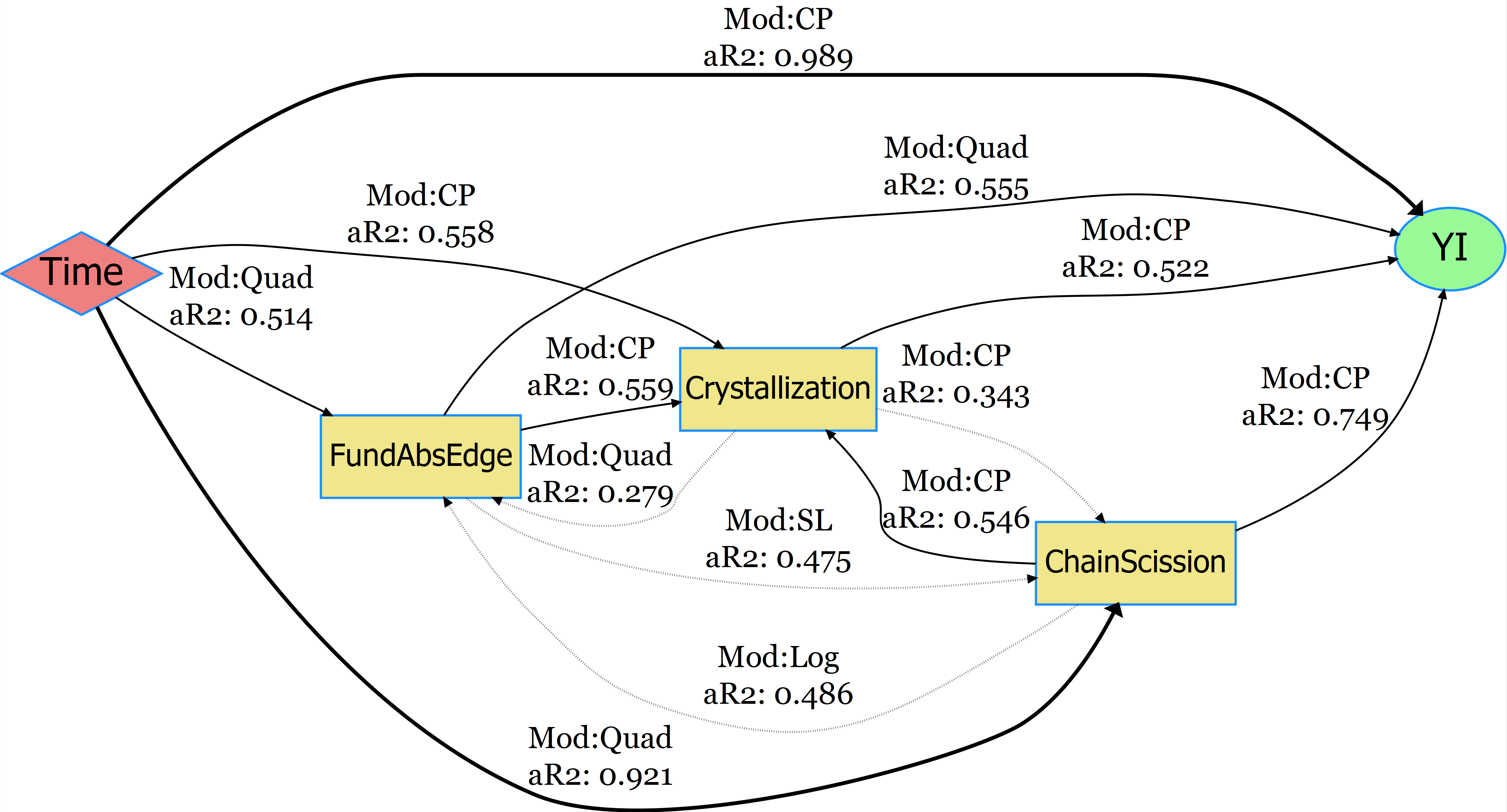

Supplement: S6 Fig — (TIFF) [file pone.0212258.s010.tiff]

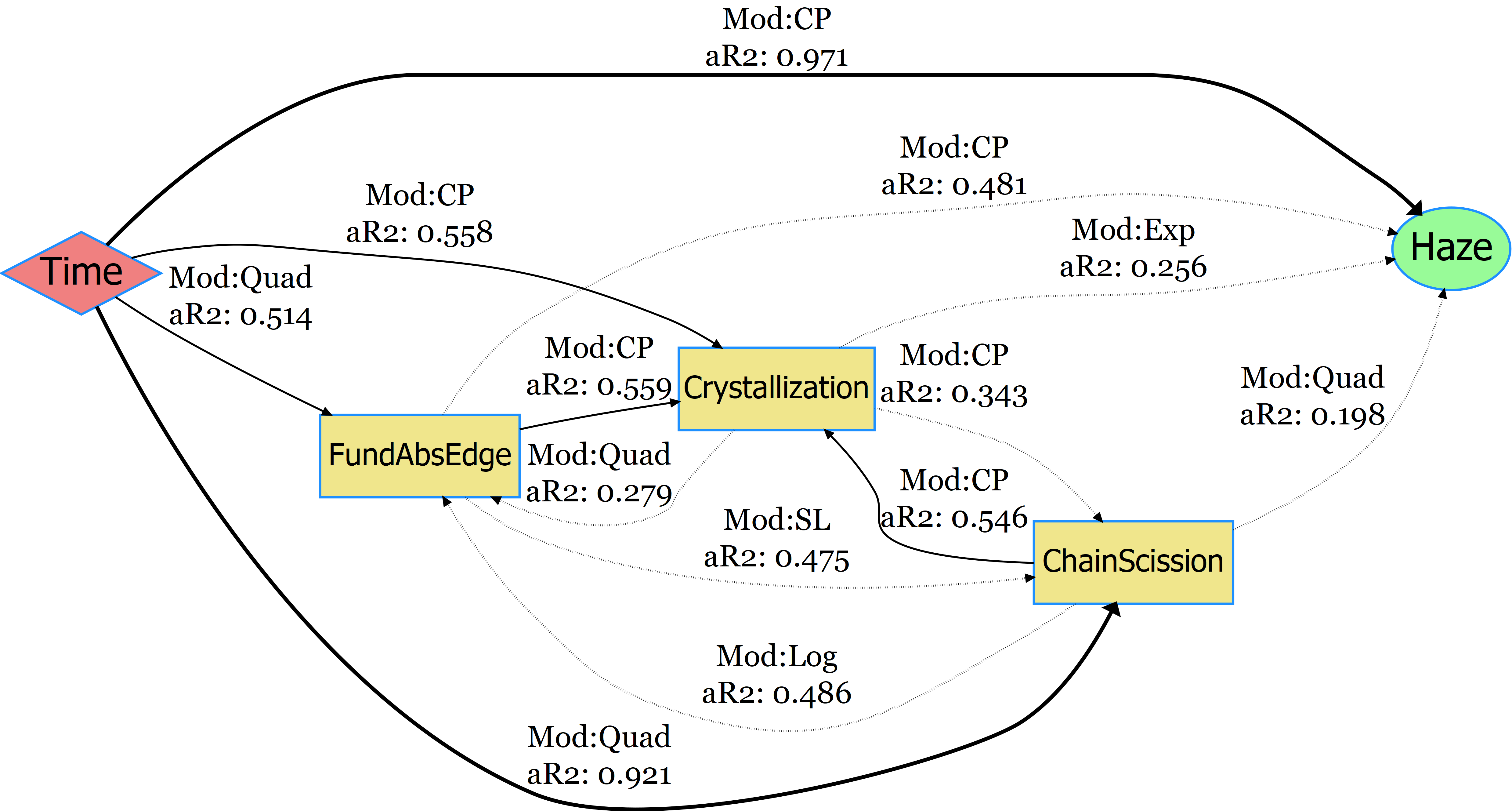

Supplement: S7 Fig — (TIFF) [file pone.0212258.s011.tiff]

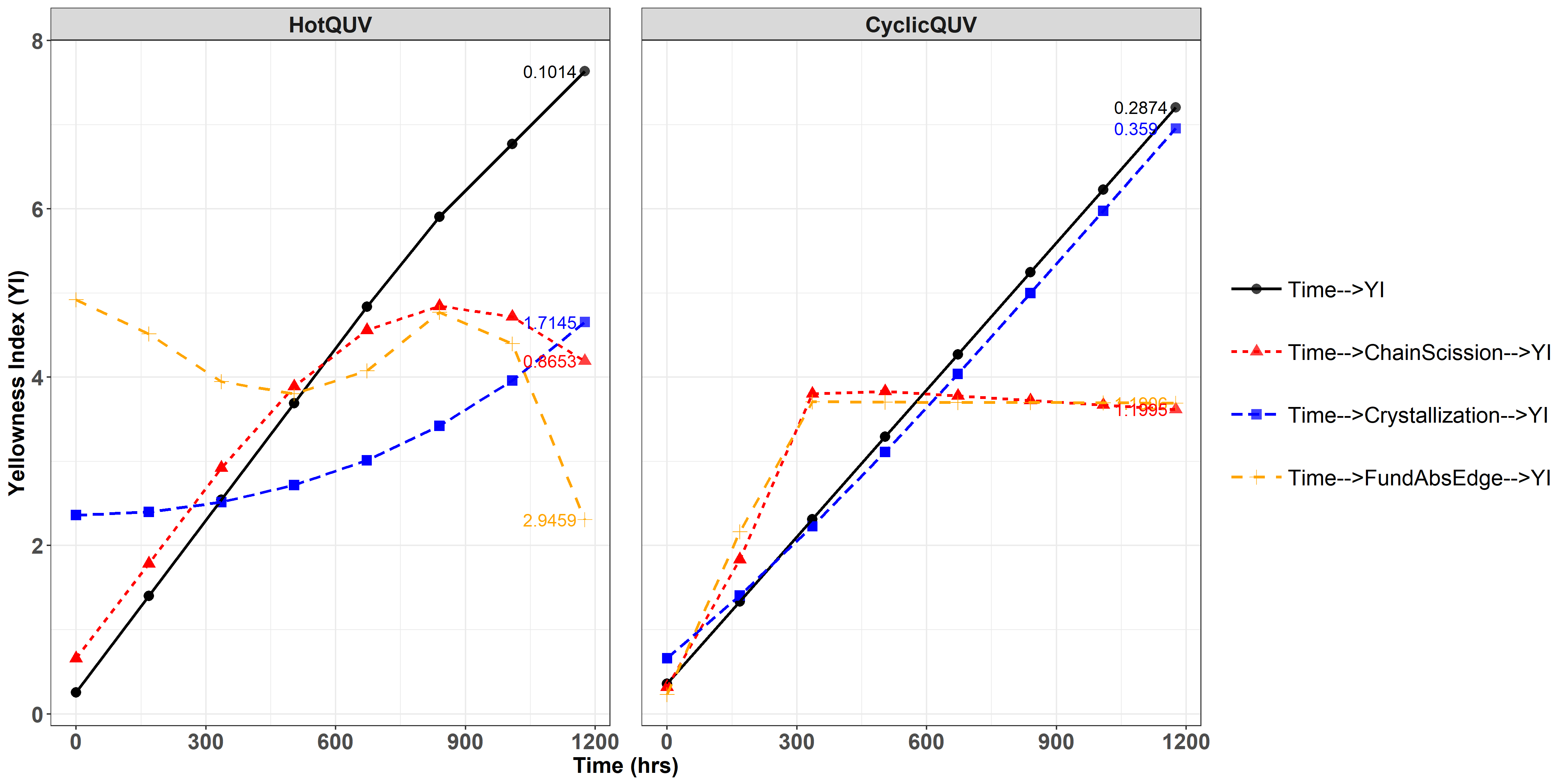

Supplement: S8 Fig — (TIFF) [file pone.0212258.s012.tiff]

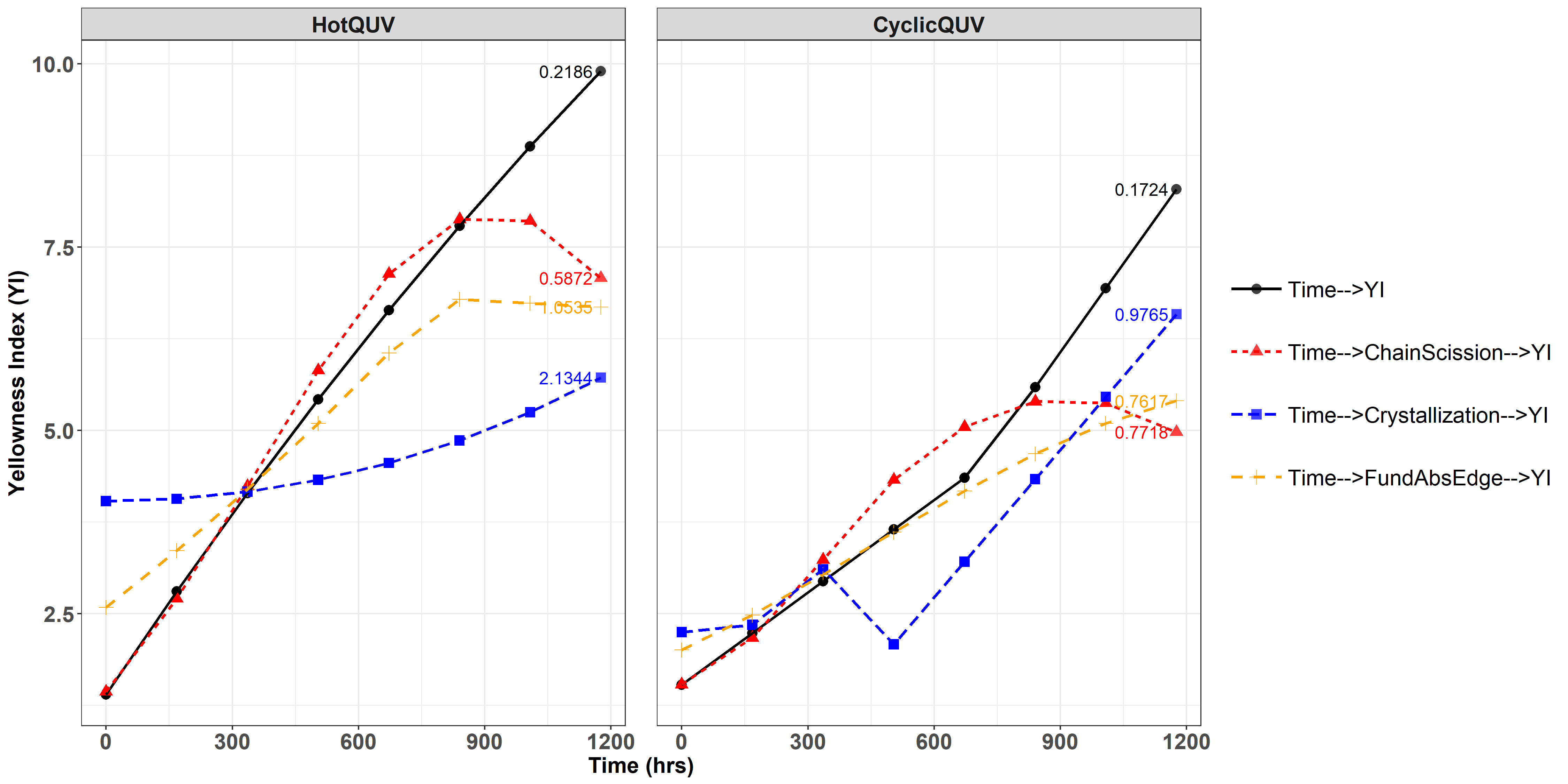

Supplement: S9 Fig — (TIFF) [file pone.0212258.s013.tiff]

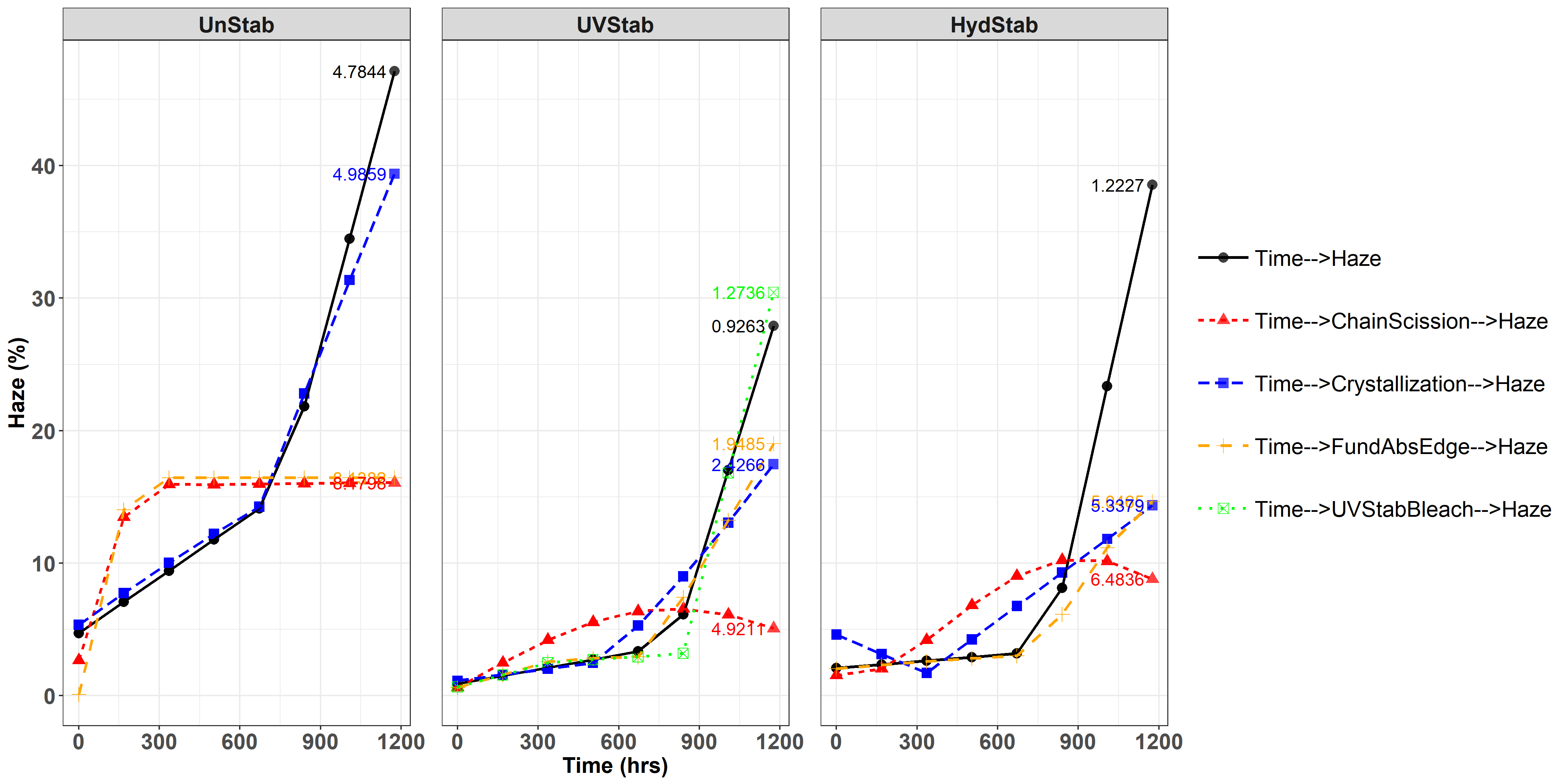

Supplement: S10 Fig — (TIFF) [file pone.0212258.s014.tiff]
